# Irradiation-Induced Upregulation of miR-711 Inhibits DNA Repair and Promotes Neurodegeneration Pathways

**DOI:** 10.3390/ijms21155239

**Published:** 2020-07-23

**Authors:** Boris Sabirzhanov, Oleg Makarevich, James P. Barrett, Isabel L. Jackson, Ethan P. Glaser, Alan I. Faden, Bogdan A. Stoica

**Affiliations:** 1 Center for Shock Trauma Anesthesiology Research, Department of Anesthesiology, University of Maryland School of Medicine, 655 W. Baltimore Street, BRB 6-015, Baltimore, MD 21201, USA; Oleg.Makarevich@som.umaryland.edu (O.M.); james.barrett@som.umaryland.edu (J.P.B.); ethang1105@gmail.com (E.P.G.); afaden@som.umaryland.edu (A.I.F.); 2 Division of Translational Radiation Sciences (DTRS), Department of Radiation Oncology, University of Maryland School of Medicine, 685 West Baltimore Street, MSTF 700-B, Baltimore, MD 21201, USA; ijackson@som.umaryland.edu; 3 VA Maryland Health Care System, Baltimore VA Medical Center, Baltimore, MD 21201, USA

**Keywords:** radiation, neuronal apoptosis, Puma, Noxa, MOMP, microRNA (miR), Rad50, Rad54l2

## Abstract

Radiotherapy for brain tumors induces neuronal DNA damage and may lead to neurodegeneration and cognitive deficits. We investigated the mechanisms of radiation-induced neuronal cell death and the role of miR-711 in the regulation of these pathways. We used in vitro and in vivo models of radiation-induced neuronal cell death. We showed that X-ray exposure in primary cortical neurons induced activation of p53-mediated mechanisms including intrinsic apoptotic pathways with sequential upregulation of BH3-only molecules, mitochondrial release of cytochrome c and AIF-1, as well as senescence pathways including upregulation of p21^WAF1/Cip1^. These pathways of irradiation-induced neuronal apoptosis may involve miR-711-dependent downregulation of pro-survival genes Akt and Ang-1. Accordingly, we demonstrated that inhibition of miR-711 attenuated degradation of Akt and Ang-1 mRNAs and reduced intrinsic apoptosis after neuronal irradiation; likewise, administration of Ang-1 was neuroprotective. Importantly, irradiation also downregulated two novel miR-711 targets, DNA-repair genes Rad50 and Rad54l2, which may impair DNA damage responses, amplifying the stimulation of apoptotic and senescence pathways and contributing to neurodegeneration. Inhibition of miR-711 rescued Rad50 and Rad54l2 expression after neuronal irradiation, enhancing DNA repair and reducing p53-dependent apoptotic and senescence pathways. Significantly, we showed that brain irradiation in vivo persistently elevated miR-711, downregulated its targets, including pro-survival and DNA-repair molecules, and is associated with markers of neurodegeneration, not only across the cortex and hippocampus but also specifically in neurons isolated from the irradiated brain. Our data suggest that irradiation-induced miR-711 negatively modulates multiple pro-survival and DNA-repair mechanisms that converge to activate neuronal intrinsic apoptosis and senescence. Using miR-711 inhibitors to block the development of these regulated neurodegenerative pathways, thus increasing neuronal survival, may be an effective neuroprotective strategy.

## 1. Introduction

Ionizing radiation (IR) is an important treatment for brain tumors [1]. Unfortunately, radiotherapy is associated with irreversible effects, including DNA damage-induced neuronal cell death [2,3,4,5], and may result in neurocognitive dysfunctions [6,7,8,9,10].

IR-induced DNA damage may initiate neuronal apoptotic pathways leading to neuronal cell death [2,3,5] via various mechanisms such as activation of p53 and caspase activation [11]. One major mechanism involves p53 as a key regulator of DNA damage-induced neuronal intrinsic apoptosis [12,13,14]. Activated p53 binds to the promoters of its target genes and upregulates the transcription of pro-apoptotic Bcl-2 family members Puma, Noxa and Bim. The role of p53 and downstream apoptotic pathways in irradiation-mediated neurotoxicity is suggested by studies showing that the absence of p53 attenuated apoptosis and volume loss after radiation therapy [15]. Furthermore, agents that downregulate the expression of pro-apoptotic Bcl-2 genes, such as valproic acid [16] and lithium [17,18], may attenuate IR-induced neuronal apoptosis and improve cognitive performance.

Akt (protein kinase B) promotes neuronal survival by phosphorylating/inactivating pro-apoptotic proteins, such as forkhead transcription factor, FoxO3, and GSK3α/β, which are essential initiators of pro-apoptotic Bcl-2 family responses [13,19,20]. Therefore, Akt activation reduces the expression of pro-apoptotic genes, such as Bim and PUMA [13]. Angiopoietin-1 (Ang-1), through activation of Akt [21,22], is neuroprotective in models of neuronal cell death, including in a DNA-damage model of neuronal apoptosis [23].

IR-induced DNA damage may also stimulate senescence pathways in adult post-mitotic neurons, including activation of p21^WAF1/Cip1^ (CDKN1A), a p53-dependent gene [24]. Even in conditions when the p53 pro-apoptotic function is not detected, sensory ganglion neurons exposed to sublethal IR show specific activation of p53/p21pathways [25].

MicroRNAs (miRs) are short noncoding RNAs that negatively regulate gene expression at the post-transcriptional level by binding to the 3′-untranslated region of target mRNAs [26]. miR-mRNA binding is mediated by Argonaute proteins within the RNA-induced silencing complex (RISC) and, contingent upon the amount of sequence complementarity, results in either cleavage of the target mRNA or reduction in its translational efficiency [27]. Although previous studies have profiled changes in brain miRs following head [28,29] or whole-body irradiation [30,31,32], their neuron-specific effects have not been investigated.

We have previously shown that miR-711 is upregulated in in vivo models of experimental traumatic brain injury (TBI) and may promote neuronal cell death due to downregulation of Akt [27] and Ang-1 [23]. Protecting neurons from loss/degeneration can alleviate the development of neurocognitive deficits after brain irradiation, but achieving this protection requires a better understanding of the molecular mechanisms involved in neuronal responses after IR [33]. We examined the mechanisms of neuronal DNA damage/repair, intrinsic apoptosis and senescence after irradiation as well as the role of miR-711 as a novel regulator of these processes.

## 2. Results

### 2.1. Anti-Apoptotic, DNA-Repair and Neuronal Marker Genes Are Downregulated while Pro-Apoptotic Genes, Pro-Senescence Genes and miR-711 Are Upregulated in the Cortex, Hippocampus and Purified Neurons after Brain Irradiation

Anti-apoptotic, DNA-repair and neuronal marker genes are downregulated while pro-apoptotic genes, pro-senescence genes and miR-711 are upregulated in the cortex, hippocampus and purified neurons after brain irradiation.

**Figure 1:**
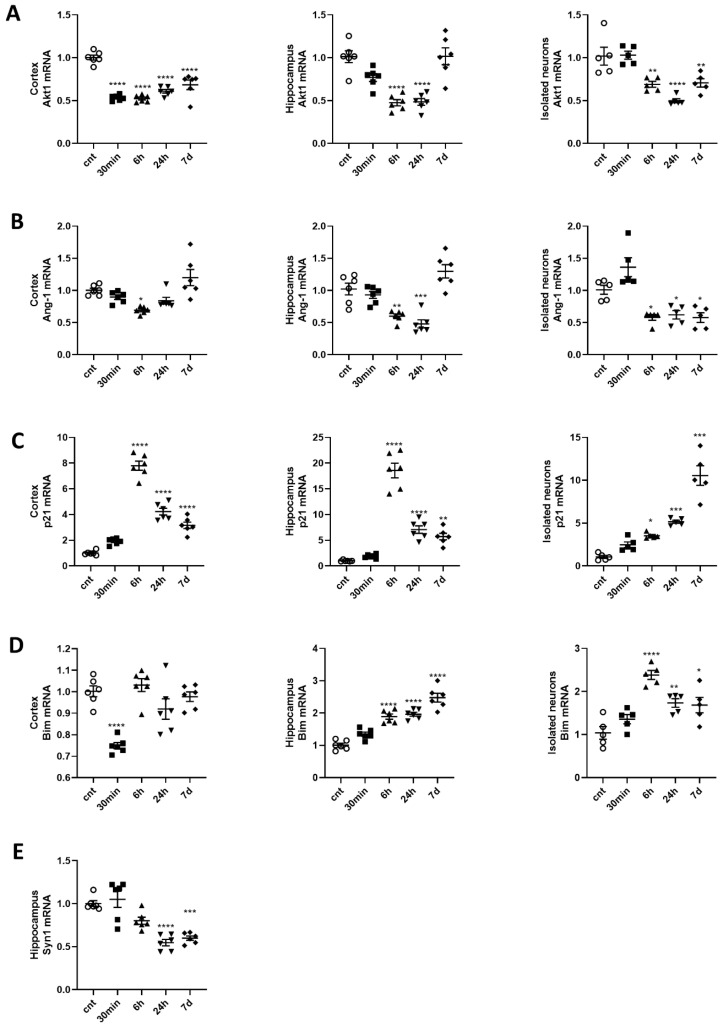
Expression of anti-apoptotic and neuronal marker genes is downregulated, while expression of pro-apoptotic and pro-senescence genes is upregulated, in the cortex, hippocampus and purified neurons after brain irradiation. Experimental rationale and details are described in Experimental Setup. Tissues and neurons were collected at 30 min, 6 h, 24 h and 7 d after 10Gy whole-brain irradiation. Total RNA was used for qPCR analysis. qPCR quantification of (**A**) Akt1 mRNAs in cortex (F (4,25) = 40.18), hippocampus (F (4,25) = 18.50) and isolated neurons (F (4,20) = 15.48); (**B**) Ang-1 mRNAs in cortex (F (4,25) = 8.438), hippocampus (F(4,25) = 20.14) and isolated neurons (F(4,20) = 15.76); (**C**) p21 mRNAs in cortex (F(4,25) = 128.4), hippocampus (F(4,25) = 82.96) and isolated neurons (F(4,20) = 44.66); (**D**) Bim in hippocampus (F(4,25) = 44.84) and isolated neurons (F(4,20) = 11.61) and (**E**) Syn1s (F(4,25) = 19.18) mRNAs in hippocampus. *n* = 6/group for tissues, *n* = 6/group for isolated neurons, with 2 technical replicates. * *p* < 0.05, ** *p* < 0.01, *** *p* < 0.001, **** *p* < 0.0001 vs. control animals non irradiated animals.

We analyzed the expression of key genes and miR-711 in the cortex and hippocampus. We also utilized isolated (purified) neurons from cortical and sub-cortical regions of the experimental animals’ brains to confirm that the observed changes in gene expression reflect neuronal changes. These targets were examined at 30 min, 6 h, 24 h and 7 d after whole-brain 10Gy exposure of male C57BL/J6 mice. Irradiation caused downregulation of Akt1 at all time points in the cortex; at 6 and 24 h in the hippocampus; and at 6 h, 24 h and 7 d in isolated neurons (Figure 1A). Ang-1 mRNA levels decreased at 6 h in the cortex; at 6 and 24 h in the hippocampus; and at 6 h, 24 h and 7 d in isolated neurons. (Figure 1B). We analyzed levels of the p53-dependent gene p21^WAF1/Cip1^ to evaluate the activation of senescence pathways. Expression of p21 was elevated in the cortex, hippocampus and isolated neurons at 6 h, 24 h and 7 d after irradiation (Figure 1C). The p53-dependent pro-apoptotic gene Bim was also upregulated in the hippocampus and isolated neurons at 6 h, 24 h and 7 d after irradiation (Figure 1D). Bim expression did not change in the cortex (data not shown). A decrease in Synapsin I (Syn1) [34], marker of synaptic degeneration and neuronal damage, was observed in the hippocampus at later time points (1 and 7d) after IR (Figure 1E).

**Figure 2:**
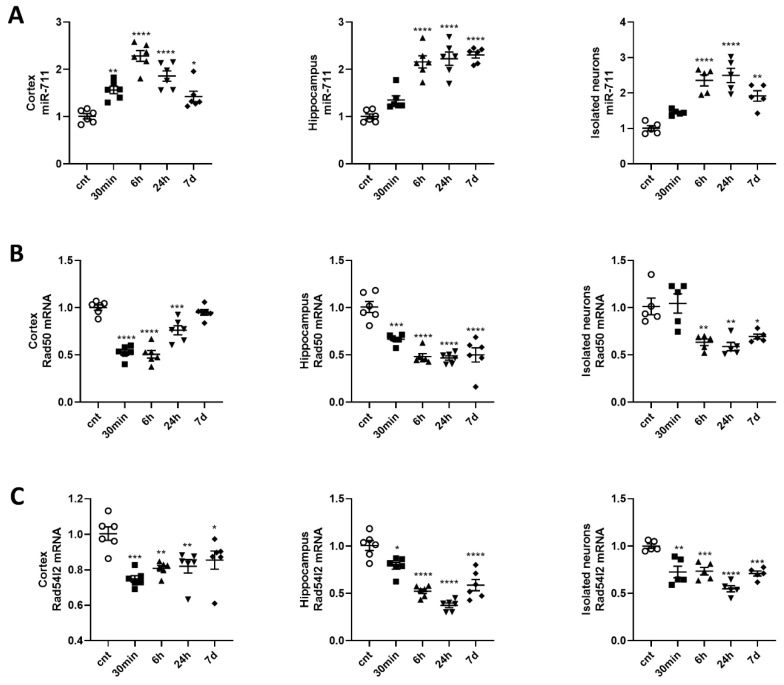
Expression of miR-711 is upregulated, while expression DNA-repair genes is downregulated, in the cortex, hippocampus and purified neurons after brain irradiation. Experimental rationale and details are described in Experimental Setup. Tissues and neurons were collected at 30 min, 6 h, 24 h and 7 d after 10Gy whole-brain irradiation. Total RNA was used for qPCR analysis. qPCR quantification of (**A**) miR-711 in cortex (F(4,25) = 24.46), hippocampus (F(4,25) = 34.3), and isolated neurons (F(4,20) = 20.88); (**B**) Rad50 mRNAs in cortex (F(4,25) = 41.65), hippocampus (F(4,25) = 23.99) and isolated neurons (F(4,20) = 10.74); (**C**) Rad54l2 mRNAs in cortex (F(4,25) = 7.513), hippocampus (F(4,25) = 35.05) and isolated neurons (F(4,20) = 17.2). *n* = 6/group for tissues, *n* = 6/group for isolated neurons, with 2 technical replicates. * *p* < 0.05, ** *p* < 0.01, *** *p* < 0.001, **** *p* < 0.0001 vs. control animals non irradiated animals.

miR-711 was upregulated in the cortex at all time points and in the hippocampus and isolated neurons at 6 h, 24 h and 7 d after IR exposure (Figure 2A–C). The upregulation of miR-711 is not reflective of a non-specific response, as another miR was downregulated after IR [35]. The DNA repair pathway molecules Rad50 and Rad54l2, novel predicted targets of miR-711, were also downregulated in the cortex, hippocampus and isolated neurons starting from 30 min and up to 7 d after IR (Figure 1B,C).

### 2.2. IR Induces Activation of DNA-Damage and p53 Pathways in Primary Cortical Neurons

**Figure 3:**
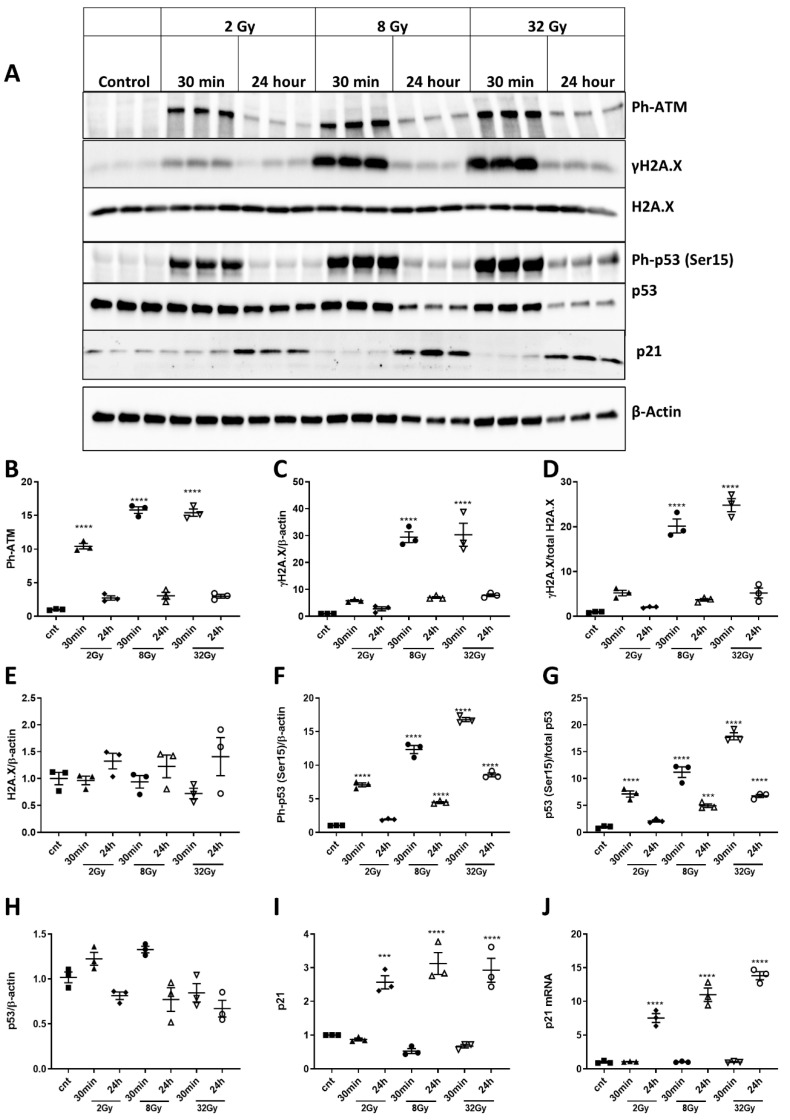
Ionizing radiation (IR) induces rapid and dose-dependent activation of DNA-damage and p53 pathways in primary cortical neurons. (**A**) Western blots for Ph-ATM(Ser 1981), γ-H2A.X, p53 and p21. (**B**) Ph-ATM (Ser1981) (F(6,14) = 246.4, *p* < 0.0001 for 30 min 2, 8 and 32Gy, compared to control; *p* = 0.0384 for 24 h 8Gy, *p* = 0.0483 for 24 h 32Gy, compared to control; *p* < 0.0001 for 24 h groups, compared to 30 min and for 30 min 8Gy, compared to 30 min 2Gy. (**C**) γ-H2A.X (F(6,14) = 47.02, *p* < 0.0001 for 30 min 8 and 32Gy compared, to control; *p* < 0.0001 for 30 min 8Gy compared to 30 min 2Gy). (**D**) γ-H2A.X normalized to total H2A.X (F 6,14) = 102.2, *p* < 0.0001 for 30 min 8 and 32Gy, compared to control; *p* < 0.0001 for 30 min 8Gy, compared to 30 min 2Gy; *p* = 0.045 for 30 min 32Gy, compared to 30 min 8Gy). (**E**) H2A.X normalized to β-actin. (**F**) Ph-p53 (F(6,14) = 351.5, *p* < 0.0001 for 30 min 2, 8 and 32Gy, and for 24 h 8 and 32Gy; *p* < 0.0001 for 8Gy compared to 2Gy and 32Gy, compared to 8Gy at 30 min; *p* = 0.0006 for 24 h 8Gy, compared to 24 h 2Gy; *p* < 0.0001 for 24 h 32Gy, compared to 24 h 8Gy; *p* < 0.0001 for 24 h 8Gy and 32Gy, compared to control). (**G**) Ph-p53 normalized to total p53 (F(6,14) = 114.5, *p* < 0.0001 for 30 min 2, 8 and 32Gy; *p* = 0.0007 for 24 h 8Gy, *p* < 0.0001 for 24 h 32Gy, compared to control; *p* = 0.0016 for 30 min 8Gy, compared to 30 min 2Gy, *p* < 0.0001 for 30 min 32Gy, compared to 30 min 8Gy; *p* = 0.0292 for 24 h 8Gy, compared to 24 h 2Gy). (**H**) p53 normalized to β-actin. (**I**) p21 (F(6,14) = 32.95, *p* = 0.0011 for 24 h 2Gy, *p* < 0.0001 for 24 h 8Gy and *p* = 0.0001 for 24 h 32Gy, compared to control). Data presented as fold change to non-irradiated control levels after normalization. *n* = 3/group; * *p* < 0.05, ** *p* < 0.01, *** *p* < 0.001, **** *p* < 0.0001 vs. control. (**J**) Neurons were collected 30 min and 24 h after 2, 8 and 32Gy irradiation. Total RNA was used for qPCR analysis. qPCR quantification of p21 mRNA. (F(6,14) = 17.7, *p* < 0.0001 for all 24 h time points, compared to control). Data presented as fold change to non-irradiated control group. *n* = 3/group; **** *p* < 0.0001 vs. control.

We measured the levels of Ph-ATM (Ser1981) [36,37], γH2A.X [38], total H2A.X, p53, Ph-p53 (Ser15) and p21 [39] by Western blot 30 min and 24 h after 2, 8 and 32Gy irradiation. All phosphorylated proteins were rapidly upregulated in a dose-dependent manner. Normalization of γ-H2A.X and Ph-p53(Ser15) to β-actin or their respective parent proteins yielded the same result. We did not observe significant increases in γ-H2A.X (Figure 3A,C,D) 30 min after 2Gy. However, levels of Ph-ATM(Ser1981) (Figure 3A,B) were elevated 30 min after 2Gy, and both Ph-ATM(Ser1981) and γ-H2A.X were significantly increased at 30 min after 8Gy. γ-H2A.X (when normalized to total H2A.X) was also higher 30 min after 32Gy vs. after 8Gy. The Ph-ATM(Ser1981) levels at 24 h were significantly decreased, compared to 30 min after all IR doses (Figure 3A,B). We did not observe significant changes in the levels of γ-H2A.X at 24 h (Figure 3A,C,D). IR had no significant effects on the level of total H2A.X at any time/IR dose (Figure 3A,E). The levels of Ph-p53(Ser15) were upregulated as early as 30 min (and at all measured time points) and were significantly decreased by 24 h in all groups (Figure 3A,F,G). IR caused dose-dependent p53 phosphorylation (2Gy vs. 8/32Gy). Ph-p53 (Ser15) levels at 24 h were decreased, compared to 30 min, but still significantly higher than control levels for 8Gy and 32Gy. We detected no IR-induced changes in total p53 (Figure 3A,H). The levels of senescence-marker p21 were also significantly upregulated at 24 h after all doses of IR, with no change at 30 min (Figure 3A,I). qPCR analysis of p21 mRNA levels matched the protein data in showing upregulation of p21 expression at 24 h after IR, compared to control Rat primary cortical neurons (RCNs) (Figure 3A,J), but, unlike the protein data, the increase of p21 expression was also dose-dependent.

### 2.3. Noxa, Puma and Bim Are Upregulated after Irradiation

**Figure 4:**
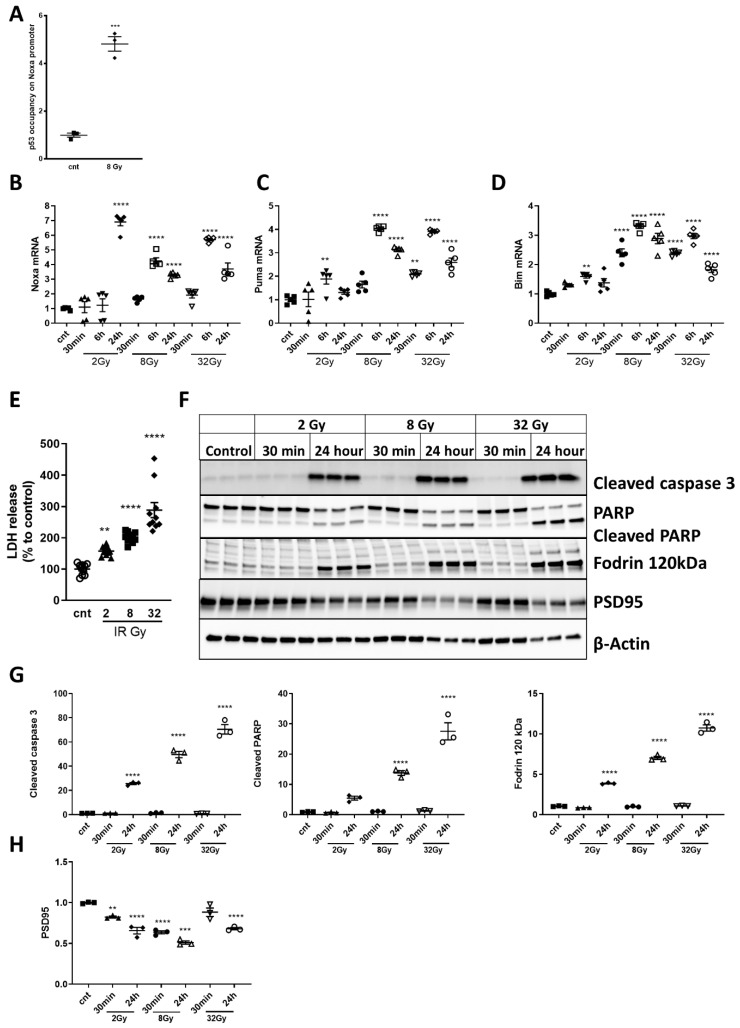
Select pro-apoptotic members of the Bcl-2 family are upregulated in irradiated primary cortical neurons. Post-ChIP qPCR to examine occupancy of p53 on Noxa promoter region (**A**). (T(4) = 2.08, *p* = 0.0001). Data presented as fold change to non-irradiated control group. *n* = 3/group. Significance assigned based on one-tailed t-test, *** *p* < 0.001 versus control Rat primary cortical neurons (RCNs). qPCR quantification of Noxa (**B**) (F(9,40) = 63.76, *p* < 0.0001 for 24 h 2Gy and for 6 h and 24 h 8 and 32Gy, compared to control), Puma (**C**) (F(9,40) = 56.47, *p* = 0.0051 for 6 h 2Gy, *p* = 0.0002 for 30 min 32Gy, and *p* < 0.0001 for 6 and 24 h 8Gy and 32Gy, compared to control), Bim (**D**) (F(9,40) = 65.12, *p* = 0.0044 for 6 h 2Gy, *p* < 0.0001 for all-time points treated with 8 and 32Gy except 30 min 8Gy, compared to control). Data presented as fold change to non-irradiated control group. *n* = 5 + /group, * *p* < 0.05, ** *p* < 0.01, *** *p* < 0.001, **** *p* < 0.0001 vs. control. Irradiation induces dose-dependent neuronal cell death and time- and dose-dependent activation of caspase-dependent apoptosis. Irradiation induces dose-dependent neuronal cell death, and time- and dose-dependent activation of caspase-dependent apoptosis in RCNs. Neurons were irradiated with 2, 8 and 32Gy. Twenty-four hours later, LDH release was measured (**E**) (F(3,36)= 7.43, *p* = 0.0175 for 2Gy, *p* < 0.0001 for 8 and 32Gy). Data expressed as a percentage of levels of non-irradiated control. *n* = 10/group, * *p* < 0.05, **** *p* < 0.0001 vs. control. Neurons were collected 30 min and 24 h after 2, 8 and 32Gy IR. Whole-cell lysates were separated by SDS-polyacrylamide gel and immunoblotted with antibodies against cleaved caspase-3, PARP, α-fodrin, PSD95 and β-actin (**F**). (**G**) Quantification of levels of cleaved caspase 3 (F(6,14) = 248.2, *p* < 0.0001 for 24 h at all doses, compared to control), PARP (F(6,14) = 79.08, *p* < 0.0001 for 24 h at 8 and 32Gy, compared to control), α-fodrin (120kDa) (F(6,14) = 553.4, *p* < 0.0001 for 24 h at all doses, compared to control) and PSD95 (**H**) (F(6,14) = 35.55, *p* = 0.0087 for 30 min 2Gy, *p* < 0.0001 at all other doses/times, except 30 min 32Gy, compared to control). *n* = 3/group, * *p* < 0.05, ** *p* < 0.01, *** *p* < 0.001, **** *p* <0.0001 vs. control.

Using ChIP analysis, we demonstrated that IR (8Gy) significantly increased the occupancy of p53 on the Noxa promoter at 6 h, compared to control (Figure 4A). qPCR analysis demonstrated a dose- and time-dependent increase in expression (peak levels at 6 h) of pro-apoptotic members of the Bcl-2 family, Noxa [40] (Figure 4B), Puma [41] (Figure 4C) and Bim [13] (Figure 4D).

### 2.4. Irradiation Induces Dose-Dependent Neuronal Cell Death and Time- and Dose-Dependent Activation of Caspase-Dependent Apoptosis

In RCNs exposed to 2, 8, and 32Gy of IR, we observed significant, dose-dependent cell death (LDH assay) at 24 h for all three IR doses (Figure 4E). Irradiated neurons displayed increased levels of apoptotic markers at 24 h, including cleaved/activated caspase-3 [42] (Figure 4F,G), cleaved PARP after 8Gy and 32Gy (Figure 4F,G) and cleaved α-fodrin [2,3,5] (Figure 4F,G). IR caused a reduction in PSD95 [43,44] levels (a marker of neurodegeneration) in all groups except 30 min after 32Gy (Figure 4F,H).

### 2.5. Irradiation CausesUupregulation of miR-711 and Downregulation of Its Target Genes Expression

**Figure 5:**
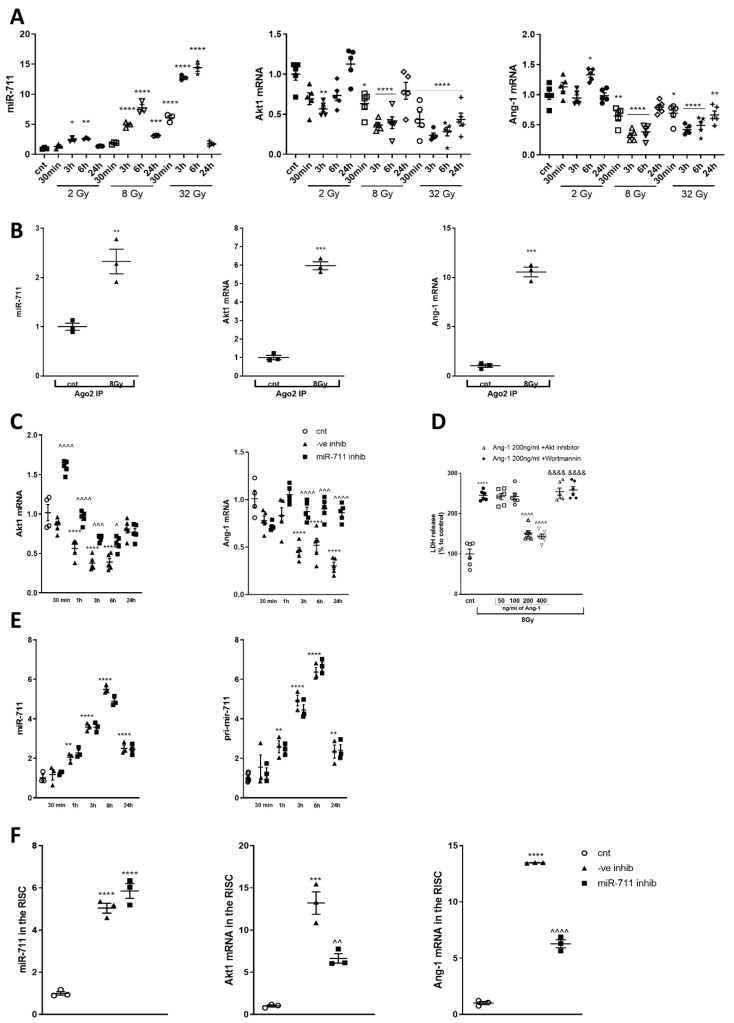
Irradiation causes upregulation of miR-711 and downregulation of its target genes expression. (**A**) qPCR quantification of miR-711 (F(12,26) = 224.6, *p* = 0.0264 for 6 h 2Gy; *p* < 0.0001 for 3 h and 6 h 8Gy, *p* = 0.0016 for 24 h 8Gy; *p* < 0.0001 for 30 min, 3 h and 6 h 32Gy, compared to control), Akt mRNA (F(12,52) = 15.04; *p* = 0.0035 for 3 h 2Gy; *p* = 0.0264 for 30 min 8Gy; *p* < 0.0001 for 6 h and 24 h 8Gy and for 32Gy at all time points, compared to control) and Ang-1 mRNA (F(12,52) = 28.39; *p* = 0.0103 for 6 h 2Gy; *p* = 0.0042 for 30 min 8Gy, *p* < 0.0001 for 3 h and 6 h 8 and 32Gy; *p* = 0.0236 for 30 min 32Gy; *p* = 0.0085 for 24 h 32Gy, compared to control). *n* = 3/group, * *p* < 0.05, ** *p* < 0.01, *** *p* < 0.001, **** *p* < 0.0001 vs. control RCNs. (**B**) Neurons were collected 6 h after 8Gy irradiation and subjected to RIP with Ago2 antibodies; samples were used for qPCR analysis. qPCR quantification of miR-711 (T(4) = 5.078, *p* = 0.0035), Akt mRNA T(4) = 20.41, *p* < 0.0001) and Ang-1 mRNA (T(4) = 8.45, *p* < 0.0001). *n* = 3/group. Significance assigned by one-tailed t-test, ** *p* < 0.01, *** *p* < 0.001 vs. control RCNs. miR-711 inhibition attenuates IR-induced downregulation of Akt and Ang-1. (**C**) RCNs were transfected with miR-711 inhibitor and miR-ve inhibitor 1 h before exposure to 8Gy. qPCR quantification of Akt mRNA (F(10,43) = 51.96, *p* < 0.0001 at 1, 3 and 6 h after IR with miR-ve inhibitor, compared to non-irradiated control; for miR-711 inhibitor, compared to miR-ve control, *p* < 0.0001 at 30 min and 1 h, *p* = 0.0010 at 3 h, *p* = 0.0356 at 6). Ang-1 mRNA (F(10,43) = 19.56, *p* < 0.0001 at 3, 6 and 24 h after IR with miR-ve inhibitor, compared to non-irradiated control; for miR-711 inhibitor, compared to miR-ve inhibitor, *p* < 0.0001 at 3 and 24 h, *p* = 0.0003 at 6 h). *n* = 4 for controls, *n* = 5/group for treatments, * *p* < 0.05, *** *p*< 0.001, vs. control; ^ *p* < 0.05, ^^^ *p* < 0.001, ^^^^ *p* < 0.0001 vs. corresponding 8Gy + miR-ve inhibitor. (**D**) RCNs were treated with recombinant Ang-1 to final concentrations 50, 100, 200 and 400 ng/mL, or with 200 ng/mL of recombinant Ang-1 and 6.25uM Akt inhibitor or 25nMWortmannin and exposed to 8Gy. LDH release was measured 24 h after irradiation (F(7,40) = 58.08, *p* < 0.0001 for IR alone vs. control; *p* < 0.0001 for 8Gy with 200 or 400 ng/mL Ang-1 vs. IR; *p* < 0.0001 for both inhibitor-containing groups vs. 8Gy with 200ng/mL Ang-1). *n* = 6/group, **** *p* < 0.0001 vs. control RCNs, ^^^^ *p* < 0.0001 vs. IR alone RCNs, &&&& *p* < 0.0001 vs. 8Gy with 200 ng/mL Ang-1. (**E**) qPCR quantification of mature miR-711 (F(10,22) = 95.15, *p* = 0.0035 at 1 h, *p* < 0.0001 at 3, 6 and 24 h after IR with miR-ve inhibitor, compared to control) and pri-mir-711 (F(10,25) = 54.32, *p* = 0.0056 at 1 h, *p* < 0.0001 at 3 and 6 h, *p* = 0.0300 at 24 h after IR with miR-ve inhibitor, compared to control). *n* = 3/group, * *p* < 0.05, ** *p* < 0.01, *** *p* < 0.001, **** *p* < 0.0001 vs. control. (**F**) Neurons were treated as describe above. Cells were collected in 3 h for RIP analysis for levels of miR-711 (F(2,6) = 112.8, *p* < 0.0001 for both treatments, compared to control), Akt1 mRNA (F(2,6) = 54.17, *p* = 0.0001 after IR with miR-ve inhibitor, compared to control, *p* = 0.0034 for miR-711 inhibitor, compared to miR-ve inhibitor) and Ang-1 mRNA (F(2,6) = 788.7, *p* < 0.0001 for all comparisons). *n* = 3/group. *** *p* < 0.001, **** *p* < 0.0001 vs. control RCNs; ^^ < 0.01, ^^^ *p* < 0.001, ^^^^ *p* < 0.0001 vs. corresponding 8Gy + miR-ve inhibitor group.

We observed a rapid and dose-dependent upregulation of miR-711 expression after 2, 8 and 32Gy (Figure 5A). 2Gy upregulated miR-711 at 3 h and 6 h. Progressively higher levels of miR-711 were observed at 3 h, 6 h and 24 h after 8Gy and at 30 min, 3 h, 6 h and 24 h after 32Gy.

Upregulation of miR-711 coincided with an opposite response (downregulation) in the expression of validated miR-711 targets Akt [27] and Ang-1 [23] (Figure 5A,C). 2Gy decreased Akt levels at 3 h, and 8Gy downregulated Akt at 3 h and 6 h, while 32Gy reduced Akt mRNA at all time points. 8Gy reduced the level of Ang-1 at 30 min, 3 h and 6 h. 32Gy downregulated Ang-1 at all time points. 2Gy did not change Ang-1 level at 30 min, 3 h or 24 h; Ang-1 was upregulated at 6 h. Both Akt and Ang-1 mRNAs levels returned to normal at 24 h after 2Gy and 8Gy. qPCR after RIP demonstrated an increase of miR-711, Akt and Ang-1 (Figure 5B) mRNAs in the RISC 6 h after 8Gy IR.

### 2.6. miR-711 Inhibition Attenuates IR-Induced Downregulation of Akt and Ang-1

In separate experiments, qPCR confirmed that 8Gy IR downregulated Akt and Ang-1 mRNAs (Figure 5C). miR-711 inhibitor increased the level of Akt mRNA at 30 min, 1 h, 3 h and 6 h after 8Gy, compared to miR-ve inhibitor (Figure 5C). [35] The IR-induced Ang-1 mRNA downregulation started at 3 h and continued up to 24 h, compared to control cells (Figure 5C). miR-711 inhibitor rescued Ang-1 mRNA at 3 h, 6 h and 24 h after IR, compared to cells treated with miR-ve inhibitor (Figure 5C). We observed significant dose-dependent attenuation of cell death after treatment with 200 and 400 ng/mL of Ang-1 at 24 h. Treatment with two Ang-1 pathway inhibitors blocked the neuroprotective effects of Ang-1 (Figure 5D). The levels of miR-711 and pri-mir-711 (a marker of miR-711 transcription) were analyzed to investigate the mechanisms of IR-induced upregulation of mature miR-711 as well as the mechanisms of action of the miR-711 inhibitor. Changes in pri-mir-711 closely paralleled the changes in mature miR-711 with increased levels of both at all time points after 8Gy IR except 30 min (Figure 5E). miR-711 inhibitors had no effect on the IR-induced increase of miR-711 and pri-miR-711 (Figure 5E). qPCR demonstrated a significant and similar IR-induced increase of miR-711, Akt (and Ang-1 (Figure 5F) mRNA in the RISC in RCNs transfected with miR-ve inhibitor. miR-711 inhibitor attenuated the IR-induced elevation of Akt (and Ang-1) (Figure 5F) mRNA in the RISC but did not change miR-711 levels in the RISC, compared to miR-ve inhibitor after 8Gy.

### 2.7. miR-711 Inhibition Attenuates Intrinsic Apoptosis After Irradiation

**Figure 6:**
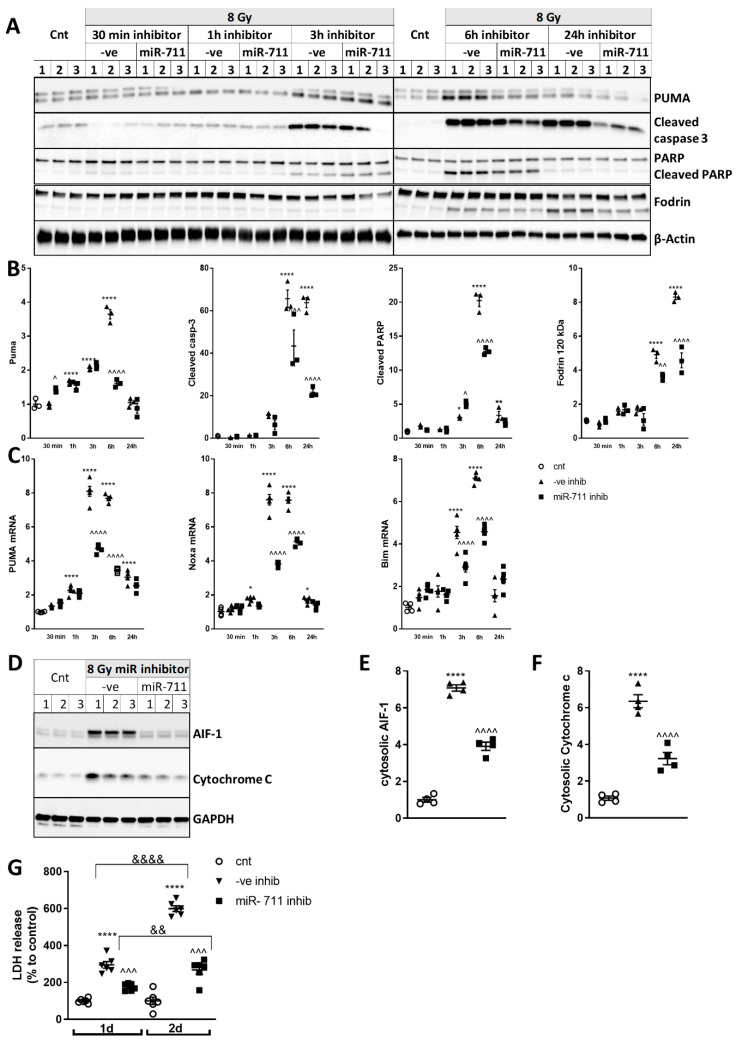
miR-711 inhibition attenuates IR-induced neuronal apoptosis. Western blot for Puma, cleaved caspase-3, PARP, α-fodrin and β-actin (**A**). (**B**) Quantification of levels of Puma (F(10,22) = 3.48, *p* = 0.0007 at 1 h, *p* < 0.0001 at 3 and 6 h after IR with miR-ve inhibitor, compared to control; for miR-711 inhibitor, compared to miR-ve inhibitor, *p* = 0.0312 at 30 min, *p* < 0.0001at 6 h), cleaved caspase-3 (F(10,22) = 5.25, *p* < 0.0001 at 6 and 24 h after IR with miR-ve inhibitor, compared to control; for miR-711 inhibitor, compared to miR-ve inhibitor, *p* = 0.0005 at 6, *p* < 0.0001 at 24 h), cleaved PARP (89kDa) (F(10,22) = 296.3, *p* = 0.0180 at 3, *p* < 0.0001 at 6, *p* = 0.0047 at 24 h after IR with miR-ve inhibitor, compared to non-irradiated control for miR-711 inhibitor, compared to miR-ve inhibitor, *p* = 0.0225 at 3, *p* < 0.0001 at 6 h), α-fodrin (120kDa) (F(10,22) = 132.9, *p* < 0.0001 at 6 and 24 h after IR with miR-ve inhibitor, compared to control for miR-711 inhibitor, compared to miR-ve inhibitor, *p* = 0.0068 at 6 h, *p* < 0.0001 at 24 h). *n* = 3/group. * *p* < 0.05, ** *p* < 0.01, *** *p* < 0.001, **** *p* < 0.0001 vs. control; ^ *p* < 0.05, ^^ *p* < 0.01, ^^^ *p* < 0.001, ^^^^ *p* < 0.0001 vs. corresponding 8Gy + miR-ve inhibitor group. qPCR quantification (**C**) of PUMA mRNA (F(10,43) = 351.7, *p* < 0.0001 at all-time points after IR with miR-ve inhibitor, compared to control; for miR-711 inhibitor, compared to miR-ve inhibitor, *p* < 0.0001 for 3 h and 6 h), Noxa mRNA (F(10,43) = 389.8, *p* = 0.0254 at 1, *p* < 0.0001 at 3 and 6, *p* = 0.0470 at 24 h after IR with miR-ve inhibitor, compared to control for miR-711 inhibitor, compared to miR-ve inhibitor, *p* < 0.0001 for 3 and 6 h) and Bim mRNA (F(10,43) = 85.04, *p* < 0.0001 at 3 and 6 h after IR with miR-ve inhibitor, compared to control for miR-711 inhibitor, compared to miR-ve inhibitor, *p* < 0.0001 at 6 and 24 h). *n* = 4 for control, *n* = 5/group for all other groups, * *p* < 0.05, **** *p* < 0.0001 vs. control; ^^^^ *p* < 0.0001 vs. corresponding 8Gy + miR-ve inhibitor group. Cytosolic fractions were used for Western blot for AIF-1, cytochrome c and GAPDH (**D**). Levels of AIF-1(**E**)(F(2,9) = 294.1, *p* < 0.0001 for all comparisons) and Cytochrome c (**F**)(F(2,9) = 4.62, *p* < 0.0001 for non-irradiated control vs. miR-ve inhibitor group and for miR-ve inhibitor vs. miR-711 inhibitor group). *n* = 3/group, **** *p* < 0.0001 vs. control RCNs; ^^^^ *p* < 0.0001 vs. 8Gy + miR-ve inhibitor group. (**G**) LDH was measured 24 h and 48 h after irradiation)(F(5,30) = 131.3, *p* < 0.0001 at 24 h and 48 after IR with miR-ve inhibitor, compared to non-irradiated control for miR-711 inhibitor, compared to miR-ve inhibitor, *p* = 0.0002 at 24 h, *p* < 0.0001 at 48 h; For 24 h vs. 48 h 8gy + miR-ve inhibitor, *p* < 0.0001; For 24 h vs. 48 h 8Gy + miR-711 inhibitor, *p* = 0.0045). *n* = 3/group. **** *p* < 0.0001 vs. control RCNs; ^^^ *p* < 0.001, ^^^^ *p* < 0.0001 vs. corresponding 8Gy + miR-ve inhibitor group; && *p* < 0.01, &&&& *p* < 0.0001 vs. equivalent treatment 24 h after 8Gy.

Western blot demonstrated that neurons transfected with miR-ve inhibitor and exposed to 8Gy show increased levels of PUMA, cleaved caspase-3, cleaved PARP-1 and cleaved Fodrin (120kDa), compared to control (Figure 6A,B). Neurons transfected with miR-ve inhibitor showed increased levels of mRNA for PUMA, Noxa and Bim at 3 h and 6 h after irradiation (Figure 6C). Subcellular fractionation revealed that 8Gy of IR caused release from the mitochondria into the cytosol of AIF-1 and cytochrome c at 6 h compared to control (Figure 6D–F). RCNs transfected with miR-711 inhibitor displayed a general attenuation of irradiation-induced changes including PUMA, cleaved/active caspase-3, cleaved PARP and cleaved α-fodrin (120kDa), compared to neurons transfected with miR-ve inhibitor (Figure 6A,B). qPCR confirmed that miR-711 inhibitor attenuated IR-induced upregulation of Puma Noxa and Bim at 3 h and 6 h, compared to miR-ve inhibitor (Figure 6C). miR-711 inhibitor attenuated IR-induced mitochondrial release of AIF-1 and cytochrome c, compared to miR-ve inhibitor (Figure 6D–F).

LDH assay demonstrated IR-induced progressive cell death up to 48 h. miR-711 inhibitor attenuated neuronal cell death at both 24 and 48 h after irradiation (Figure 6G). Transfection of RCNs with miR-711 inhibitor or miR-ve inhibitor did not change LDH release in non-irradiated cells (data not shown).

### 2.8. miR-711 Inhibition Attenuates IR-Induced DNA Damage Markers, p53 Activation, and Neuronal Apoptosis and Senescence Markers

**Figure 7:**
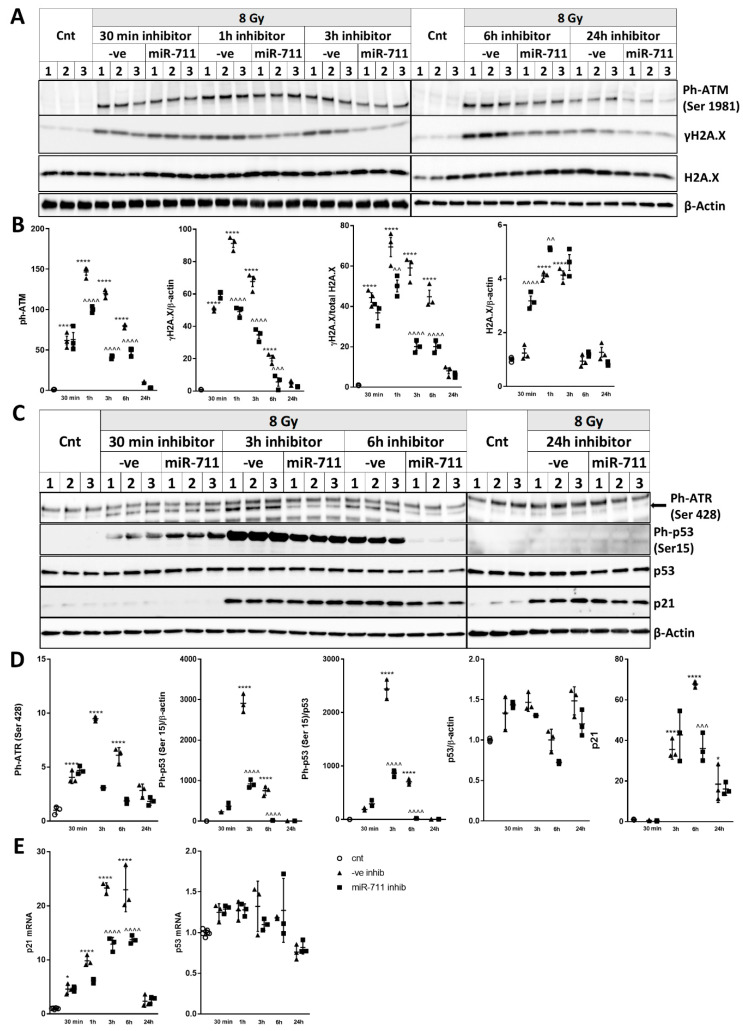
miR-711 inhibition attenuates IR-induced DNA damage markers, p53 activation, and neuronal apoptosis and senescence markers. Western blot for Ph-ATM(Ser1981), γ-H2A.X and H2A.X (**A**). (**B**) Ph-ATM(Ser1981) (F(10,22) = 173, *p* < 0.0001 at 30 min, 1 h, 3 h and 6 h after IR with miR-ve inhibitor, compared to control for miR-711 inhibitor, compared to miR-ve inhibitor, *p* < 0.0001at 1 h and 3 h, *p* = 0.0001 at 6 h), γH2A.X normalized to β-actin (F(10,22) = 281.7, *p* < 0.0001 for all time points except 24 h after IR with miR-ve inhibitor, compared to non-irradiated control; for miR-711 inhibitor, compared to miR-ve inhibitor, *p* < 0.0001 for 1 h and 3 h, *p* = 0.0005 for 6 h), γH2A.X normalized to H2A.X (F(10,22) = 73.18, *p* < 0.0001 for all time points except 24 h after IR with miR-ve inhibitor, compared to control for miR-711 inhibitor, compared to miR-ve inhibitor, *p* = 0.0017 at 1 h, *p* < 0.0001 at 3 h and 6 h). H2A.X (F(10,22) = 138.8, *p* < 0.0001 for 1 h and 3 h after IR with miR-ve inhibitor, compared to control; for miR-711 inhibitor, compared to miR-ve inhibitor, *p* < 0.0001 for 1 h and 3 h after IR with miR-ve inhibitor, compared to control; for miR-711 inhibitor, compared to miR-ve inhibitor, *p* < 0.0001 at 30 min, *p* = 0.0029 at 1 h). Western blots for Ph-ATR(Ser428), Ph-p53(Ser15), p53, p21 and β-actin (**C**). (**D**) Ph-ATR(Ser428)(F(8,18) = 108.4, *p* < 0.0001 at 30 min, 3 h and 6 h, *p* = 0.0017 at 24 h after IR with miR-ve inhibitor, compared to control; for miR-711 inhibitor, compared to miR-ve inhibitor, *p* < 0.0001 at 3 h and 6 h), Ph-p53(Ser15) normalized to β-actin (F(8,18) = 302, *p* < 0.0001 at 3 h and 6 h after IR with miR-ve inhibitor, compared to control; for miR-711 inhibitor, compared to miR-ve inhibitor, *p* < 0.0001 at 3 h and 6 h), Ph-p53(Ser15) normalized to p53(F(8,18) = 392.6, *p* < 0.0001 at 3 h and 6 h after IR with miR-ve inhibitor, compared to control; for miR-711 inhibitor, compared to miR-ve inhibitor, *p* < 0.0001 at 3 h and 6 h), p21(F(8,18) = 45.58, *p* < 0.0001 at 3 h and 6 h, *p* = 0.0410 at 24 h after IR with miR-ve inhibitor, compared to control; for miR-711 inhibitor, compared to miR-ve inhibitor, *p* = 0.0001 at 6 h). *n* = 3/group. qPCR quantification of p21 and p53 mRNAs (**E**). p21 mRNA (F(10,25) = 111, *p* = 0.0342 at 30 min, *p* < 0.0001 at 3 h and 6 h after IR with miR-ve inhibitor, compared to control; for miR-711 inhibitor, compared to miR-ve inhibitor, *p* < 0.0001 at 3 h and 6 h), p53 mRNA (F(10,25) = 5.062, no significant changes). *n* = 3/group. * *p* < 0.05, **** *p* < 0.0001 vs. control, ^^^ *p* < 0.001, ^^^^ *p* < 0.0001 vs. corresponding 8Gy + miR-ve inhibitor group. miR-711 inhibition attenuates irradiation-induced downregulation of DNA repair molecules Rad50 and Rad54l2.

Levels of Ph-ATM(Ser1981), γH2A.X, total H2A.X, Ph-ATR(Ser428), Ph-53(Ser15) and p21 were analyzed at multiple time points. 8Gy irradiation caused rapid (30 min) phosphorylation of ATM(Ser1981), which lasted at least 6 h. miR-711 inhibitor attenuated phosphorylation of ATM at 1 h, 3 h and 6 h (Figure 7A,B). After 8Gy IR, γH2A.X was upregulated at 30 min, 1 h, 3 h and 6 h, and miR-711 inhibitor attenuated IR-induced γH2A.X at 1 h, 3 h and 6 h (Figure 7A,B), whether normalized to β-actin or to total H2A.X. Neurons transfected with miR-ve inhibitor showed changes in the levels of total H2A.X, though only at 1 h and 3 h. Administration of miR-711 inhibitor upregulated levels of total H2A.X at 30 min, 1 h and 3 h after IR; at 30 min and 1 h, it caused higher H2A.X compared to miR-ve inhibitor (Figure 7A,B). Ph-ATR(Ser428) levels were increased after IR with miR-ve inhibitor up to 6 h, as were Ph-p53(Ser15) levels at 3 h and 6 h; and p21 levels at 3h3 h, 6 h and 24 h compared to control (Figure 7C,D). miR-711 inhibitor attenuated post-IR upregulation of Ph-ATR(Ser428) at 3 h and 6 h; Ph-p53(Ser15) at 1 h, 3 h and 6 h; and p21 at 6 h, compared to miR-ve inhibitor (Figure 7C,D). Ph-p53(Ser15) showed the same result whether normalized to β-actin or total p53. p21 mRNA was upregulated after IR with miR-ve inhibitor up to 6 h compared to control. Administration of miR-711 inhibitor attenuated the IR-induced upregulation of p21 expression (Figure 7E). Neither IR nor miR-711 inhibitor altered the level of total p53 protein or mRNA, suggesting that p53 is not a direct target of miR-711 (Figure 7C–E).

### 2.9. miR-711 Inhibition Attenuates Irradiation-Induced Downregulation of DNA Repair Molecules Rad50 and Rad54l2

**Figure 8:**
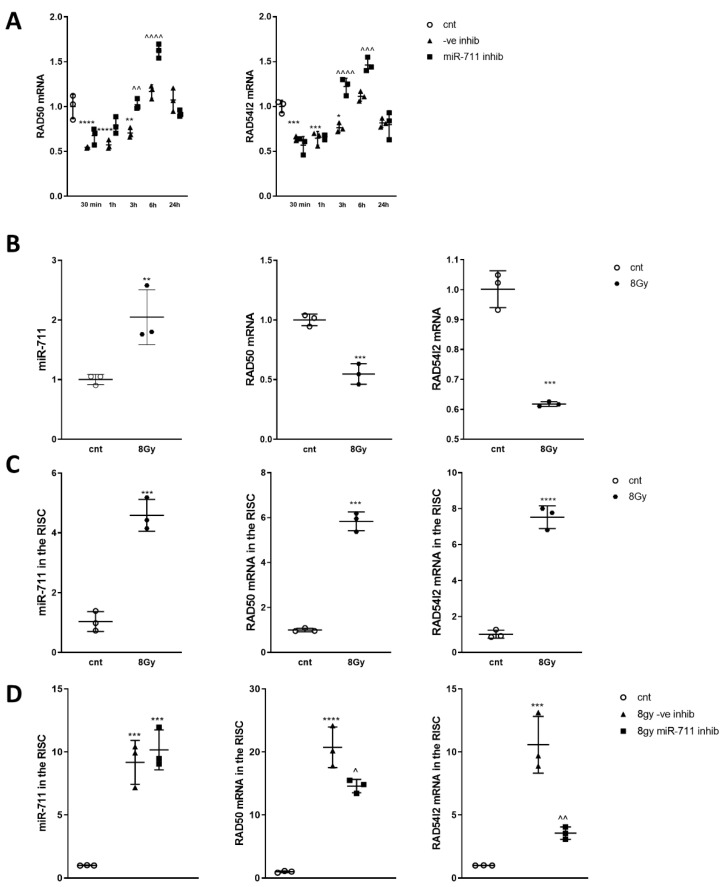
miR-711 inhibition attenuates irradiation-induced downregulation of DNA repair molecules Rad50 and Rad54l2. qPCR quantification of RAD50 and RAD54l2 (**A**). Rad50 mRNA (F(10,22) = 43.49, *p* < 0.0001 at 30 min and 1 h, *p* = 0.0082 at 3 h after IR with miR-ve inhibitor, compared to control; for miR-711 inhibitor, compared to miR-ve inhibitor, *p* = 0.0039 at 3 h, *p* < 0.0001 at 6 h). Rad54l2 mRNA (F(10,22) = 39.1, *p* = 0.0005 at 30 min, *p* = 0.006 at 1 h, *p* = 0.0396 at 3 h after IR with miR-ve inhibitor, compared to control; for miR-711 inhibitor, compared to miR-ve inhibitor, *p* < 0.0001 at 3 h, *p* = 0.0007 at 6 h). *n* = 3/group for all groups, * *p* < 0.05, ** *p* < 0.01, *** *p* < 0.001, **** *p* < 0.0001 vs. control; ^ *p* < 0.05, ^^ *p* < 0.01, ^^^ *p* < 0.001, ^^^^ *p* < 0.0001 vs. corresponding 8Gy + miR-ve inhibitor group. RCNs were exposed to 8Gy and, after 3 h, cells were collected and lysed. One part of the lysate from each sample was used for RNA isolation and qPCR analysis for levels of miR-711, RAD50 and RAD54l2 (**B**). miR-711 (T(4) = 3.856, *p* = 0.0091), Rad50 mRNA (T(4) = 7.944, *p* = 0.0007), Rad54l2 mRNA (T(4) = 0.74, *p* = 0.0002). The other part was subjected to RIP using Ago2 antibodies, followed by qPCR analysis for levels of miR-711, RAD50 and RAD54l2 in the RISC (**C**). miR-711 (T(4) = 9.77, *p* = 0.0003); Rad50 mRNA (T(4) = 9.55, *p* < 0.0001); Rad54l2 mRNA (T(4) = 16.8, *p* < 0.0001). *n* = 3/group. Significance assigned by one-tailed t-test, *** *p* < 0.001, **** *p* < 0.0001 vs. control RCNs. This experiment was repeated with miR-711 inhibitor and miR-ive inhibitor. qPCR analysis for levels of miR-711, Rad50 and Rad54l2 (**D**): miR-711 (F(2,6) = 41.13, *p* = 0.0008 after IR with miR-ve inhibitor, compared to control), Rad50 mRNA (F(2,6) = 79.83, *p* < 0.0001 after IR with miR-ve inhibitor, compared to control; for miR-711 inhibitor, compared to miR-ve inhibitor, *p* = 0.0200), Rad54l2 mRNA (F(2,6) = 41.51, *p* = 0.0003 after IR with miR-ve inhibitor, compared to non-irradiated control; for miR-711 inhibitor, compared to miR-ve inhibitor, *p* = 0.0016). *n* = 3/group, *** *p* < 0.001, **** *p* < 0.0001 vs. control; ^ *p* < 0.05, ^^ *p* < 0.01 vs. corresponding 8Gy + miR-ve inhibitor group.

IR induced downregulation of Rad50 and Rad54l2 up to 3 h (Figure 8A), and these changes were attenuated by miR-711 inhibitor compared to miR-ve inhibitor. miR-711 was increased in RCNs after IR, coinciding with downregulation of its predicted targets Rad50 and Rad54l2 (Figure 8B). We detected significant upregulation of both miR-711 and its targets, Rad50 and Rad54l2, in the RISC after IR alone (Figure 8C) and after IR in RCNs transfected with miR-ve inhibitor, compared to controls. The miR-711 inhibitor attenuated the IR-induced elevation of Rad50 and Rad54l2 in the RISC without affecting the level of miR-711 compared to the miR-ve inhibitor (Figure 8D).

We also examined the effect of IR and miR-711 inhibition on other predicted miR-711 target genes Ercc2, HDAC1, HDAC2 and HDAC4, which may regulate DNA-damage responses. Expression of these genes was rapidly downregulated by 8Gy IR and returned to control levels at 6 h, but miR-711 inhibitor did not rescue their downregulation (Supplementary Figure S1).

**Figure 9:**
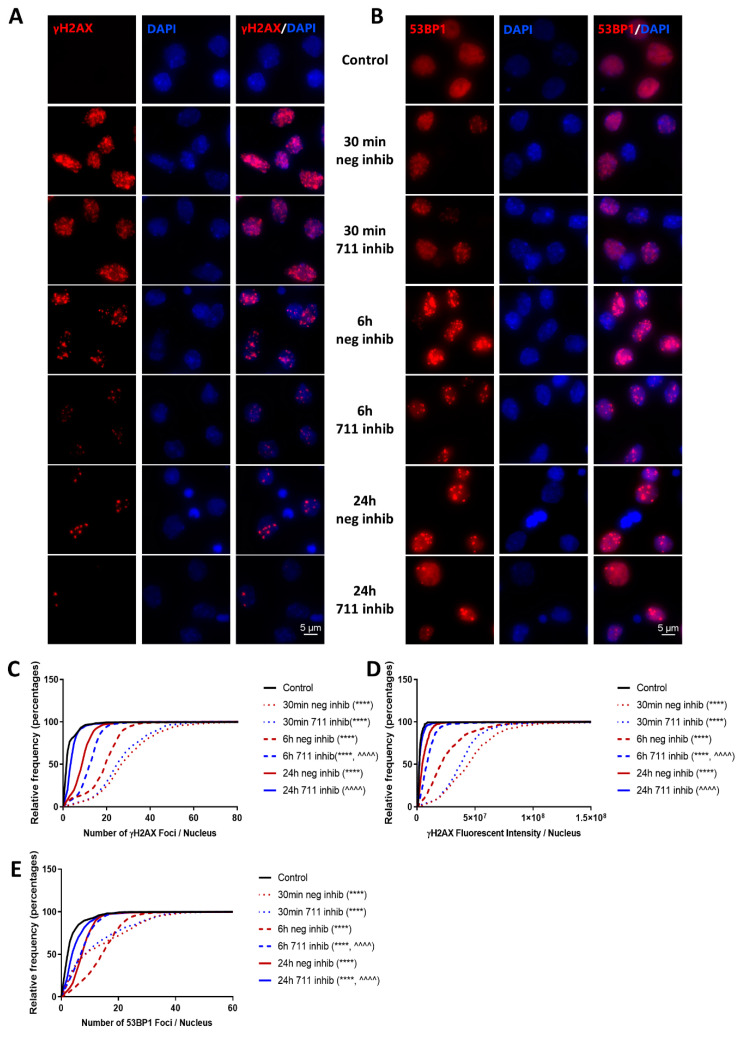
Representative microscopy images from 30 min, 6 and 24 h of RCNs stained for γ-H2A.X (red), and DAPI (blue) (**A**). Representative microscopy images from 30 min, 6 h and 24 h of RCNs stained for 53BP1 (red), and DAPI (blue) (**B**). Data were calculated and plotted for all fields together as a cumulative frequency distribution without binning for γ-H2A.X foci count (**C**) and nuclear staining intensity (**D**). Data were calculated and plotted for all fields together as a cumulative frequency distribution for 53BP1 foci count (**E**). For all three parameters (L,M,*n*), samples treated with 8Gy + miR-ve inhibitors were significantly different from controls at all time points ( *p* < 0.0001, G:H(7)= 2257, H:H(7) = 2588, I:H(7) = 1041). Irradiated samples transfected with miR-711 inhibitor were significantly different from those transfected with miR-ve inhibitors at 6 h and 24 h for all parameters ( *p* < 0.0001) but not at 30 min.

We evaluated the effect of miR-711 inhibition on DNA repair mechanisms by quantitative analysis of accumulated DNA damage based on γH2A.X and 53BP1 foci formation and progression after 8Gy irradiation. IR significantly increased γH2A.X foci number and signal intensity per nucleus (Figure 9A,C,D) and elevated 53BP1 foci number per nucleus (Figure 9B,E) at 30 min, 6 h and 24 h in miR-ve inhibitor-transfected samples compared to the non-irradiated control, shifting the cell population signal intensity distribution curve to the right; these changes were progressively attenuated with time. At 30 min, there was no significant difference between the irradiated groups, but by 6 h (and persisting through 24 h), miR-711 inhibitor led to a significant decrease in all three parameters, compared to miR-ve inhibitor, pushing the cell population distribution curve to the left.

### 2.10. Effect of miR-711 Inhibition on miR-23a-3p Expression

We have recently reported that IR downregulates miR-23a-3p, a pro-survival microRNA [35]. We examined the effect of miR-711 inhibition on the level of neuronal miR-23a-3p as well the effect of miR-23a-3p mimic on the level of miR-711 after IR exposure. qPCR confirmed that miR-711 inhibitor did not attenuate the IR (8Gy)-induced decrease of miR-23a-3p (Supplementary Figure S2A). We also examined the effect of miR-23a-3p mimic on the level of miR-711 after IR exposure. qPCR demonstrated that miR-23a-3p mimic did not attenuate the IR (8Gy)-induced increase of miR-711 (Supplementary Figure S2B). Finally, we examined the presence of additive neuroprotective effects following co-transfection of neurons with both miR-711 inhibitor and miR-23a-3p mimic. To avoid toxic and/or off-target effects [45] due to transfection with a high concentration of miR modulators, for the combined treatment we decreased the concentration of miR-711 inhibitor and miR-23a-3p mimic to 25 nM each (half of the concentration used for the individual interventions) in order to keep the total concentration of oligonucleotides at 50 nM across treatments. LDH assay demonstrated that miR-711 inhibitor, as well as miR-23a-3p mimic, attenuated neuronal cell death at 24 h after irradiation (8Gy). Co-transfection of RCNs with half-concentration miR-711 inhibitor and miR-23a-3p mimic was more neuroprotective than transfection with full-concentration miR-23a-3p mimic and as neuroprotective as full-concentration miR-711 inhibitor (Supplementary Figure S2C).

## 3. Discussion

We observed that X-ray exposure in RCNs activated DNA damage responses with sequential phosphorylation/activation of ATM(Ser1981), γH2A.X and Ph-p53(S15) (Figure 3). A key intrinsic apoptosis pathway involves p53 binding to and transactivating the promoters of pro-apoptotic Bcl-2 family members, such as Puma, Noxa and Bim [46,47,48], a mechanism confirmed by our detection of p53 interaction with the Noxa promoter and IR-induced upregulation of the p53-dependent genes, Noxa, Puma and Bim (Figure 4).

We showed that the IR-induced upregulation of pro-apoptotic Bcl2 proteins (Figure 1) is followed by mitochondrial outer membrane permeabilization (MOMP) [49] with cytosolic release of Cytochrome c (Figure 6) and AIF-1 [40], thus activating the intrinsic apoptosis pathway [42]. IR subsequently resulted in activation of caspase-3 and cleavage of its substrates PARP and α-fodrin—markers of neuronal apoptosis (Figure 4) [2,3,5], as well as downregulation of PSD95 (Figure 4), indicating synaptic degeneration and neuronal damage [43,44].

Our data suggest that irradiation-dependent stimulation of neuronal p53 pro-apoptotic pathways is underpinned by two mechanisms, p53 phosphorylation/activation (Figure 3) and inhibition of pro-survival Akt pathways. The pro-survival molecule Akt suppresses transcription of Bim and PUMA [13] and may counterbalance p53-mediated apoptosis. We confirmed the pro-survival role of the PI3K/Akt pathway following neuronal IR by showing that Ang-1 attenuates IR-induced neuronal apoptosis, while inhibition of the PI3K/Akt/Ang-1 pathway blocks the protective effect of Ang-1 (Figure 4). Therefore, IR-induced changes in miR expression affecting either mechanism may play an essential role in regulation of apoptosis [50].

Using RCNs and in vivo models of TBI, we previously reported that miR-711 elevation leads to apoptosis by downregulation of targets in the pro-survival PI3K/Akt pathway: Akt1 [27] and Ang-1 [23]; Ang-1 exerts an anti-apoptotic effect by promoting Akt activation [23]. The current studies show that IR induced upregulation of neuronal pro-apoptotic miR-711 along with decreases in Akt and Ang-1 [35]. The concurrent detection of increased levels of miR-711, Akt1 and Ang-1 in the RISC suggest that the miR-711 upregulation is responsible for the IR-induced downregulation of Akt and Ang-1 (Figure 5). Accordingly, miR-711 inhibitors decreased levels of Akt1 and Ang-1 mRNAs in the RISC, thus elevating cellular Akt1 and Ang-1 (Figure 5) with concurrent reduction of Puma, Noxa and Bim, inhibition of MOMP [49] and attenuation of cytosolic release of AIF-1 and cytochrome c [40,51] (Figure 6). Ultimately, miR-711 inhibitors also reduced activation of caspases and cleavage of their protein substrates PARP and α-fodrin (Figure 6), thus proving them capable of attenuating IR-induced neuronal apoptosis.

We have recently reported that IR induced downregulation of pro-survival neuronal miR-23a-3p [35], and our current data demonstrate that the IR-induced miR-711 elevation and miR-23a-3p decline are independent processes. However, the IR-induced miR-711 and miR-23a-3p changes converge on the same key pro-apoptotic BH3-only molecules and by different mechanisms induce their activation. Importantly, the current studies also revealed that co-transfection with miR-711 inhibitor and miR-23a-3p mimic (each at half-concentration) was more neuroprotective than single transfection (full concentration) with miR-23a-3p mimic and as effective as miR-711 inhibitor (full concentration). These results may suggest that IR-induced miR-23a-3p and miR-711 changes trigger complementary mechanisms to initiate neuronal apoptotic pathways and that combined targeting of these microRNAs may have additive neuroprotective effects.

Surprisingly, we observed that inhibition of miR-711 also decreases upstream markers of IR-induced DNA-damage responses, such as Ph-ATM(Ser1981), γH2A.X, Ph-ATR(Ser428) and Ph-p53(Ser15) (Figure 7). The attenuation of IR-induced DNA damage markers and p53 activation by miR-711 inhibition may indicate that miR-711 contributes to neuronal apoptosis not only through inhibition of Ang-1/Akt but also through modulation of DNA damage responses upstream of p53 activation.

IR causes chromosomal double-strand breaks (DSB), which trigger the DNA damage response (DDR) involving activation of DNA damage kinase ATM followed by generation of γH2A.X, which acts as a docking station, recruiting repair factors such as 53BP1 to form IR-induced foci (IRIF), also known as DNA repair foci [52]. In non-injured neurons, γH2A.X is only weakly detected, whereas 53BP1 is widely expressed with a homogenous/diffuse nuclear staining pattern. After irradiation, the nuclear distribution of both γH2A.X and 53BP1 becomes highly concentrated, reflecting the presence of γH2A.X at DSB sites and the recruitment of 53BP1 to IRIF [53]. Furthermore, a quantitative association exists between γH2A.X foci formation and DSBs after IR exposure [53]; namely, γH2A.X and 53BP1 foci are markers of DNA breaks, and the decline of the number of γH2A.X and 53BP1 foci is an indicator of DNA repair [54].

Two molecules involved in DNA repair—Rad50, crucial for the activity of double-strand break repair nuclease MRE11 [55,56,57,58], and Rad54l2, a member of the SNF2-like family of proteins involved in chromatin remodeling, DNA repair and homologous recombination [59,60]—are novel predicted miR-711 targets. We demonstrated that rapid IR-mediated downregulation of neuronal Rad50 and Rad54l2 coincides with miR-711 upregulation (Figure 2). Furthermore, irradiation increased levels of miR-711, Rad50 and Rad54l2 in the RISC (Figure 8). Importantly, administration of the miR-711 inhibitor attenuated these changes, demonstrating that miR-711 is a negative regulator of Rad50 and Rad54l2 (Figure 8). IR induced rapid formation of γH2A.X and 53BP1 foci followed by a DNA-repair-driven progressive decline. Importantly, inhibition of miR-711 accelerates the elimination of γH2A.X and 53BP1 foci (Figure 9) consistent with an enhancement of DNA repair—significant changes were detected at 6 h and 24 h but not at 30 min after irradiation, indicative of miR-711 inhibition acting on secondary processes and not on the primary DNA damage.

DNA damage repair defects that lead to persistent DNA breaks are associated with activation of the neuronal p53/p21 senescence pathways and ultimately neurodegeneration [61]. In various cells including neurons, the IR-induced senescent phenotype involves a positive feedback loop characterized by p21^WAF1/Cip1^-dependent mitochondrial dysfunction with ROS generation and further DNA damage [62]. DNA damage in mature postmitotic neurons may cause a dysfunctional senescent-like state displaying pro-oxidative/inflammatory changes and heterochromatinization, with p21^WAF1/Cip1^ acting as a necessary signal transducer [63]. We demonstrate that miR-711 inhibitor administration results in a reduction of IR-induced p21^WAF1/Cip1^ elevation, suggestive of an attenuation of senescence mechanisms triggered by neuronal irradiation.

Our observations support a model in which in the immediate aftermath of IR, ATM initiates the DDR via formation of γH2A.X/53BP1-containing IRIF. In the ensuing hours, upregulation of miR-711 reduces expression of Rad50/Rad54l2, which may decrease the effectiveness of DNA repair, delaying DSB resolution and resulting in persistent ATM signaling that strengthens the activation of p53-dependent apoptotic and senescence pathways. In contrast, administration of miR-711 inhibitor leads to elevation of Rad50 and Rad54l2, which enhance the repair of radiation-induced DNA damage. The improved clearance of DSB, as evidenced by the more rapid reduction in IRIF, may contribute to attenuation of p53-dependent apoptotic and senescence pathways and improved neuronal survival.

To elucidate the mechanisms of IR-induced neurodegeneration, we have examined key elements of these mechanisms in an in vivo model of brain irradiation. Thus, we demonstrated that IR induced miR-711 and caused downregulation of Akt1 and Ang-1, DNA repair genes Rad50 and Rad54l2, as well as induction of pro-senescence gene p21^WAF1/Cip1^ in the cortex and hippocampus. The magnitude and duration of these changes were especially prominent in the hippocampus, where we also observed robust upregulation of the pro-apoptotic gene Bim and downregulation of neuronal marker Syn1. Significantly, we demonstrated that a similar pattern of molecular changes occurs in neurons isolated from the irradiated brains; some genes, including p21^WAF1/Cip1^, displayed their highest and most persistent changes in the purified neurons.

**Figure 10:**
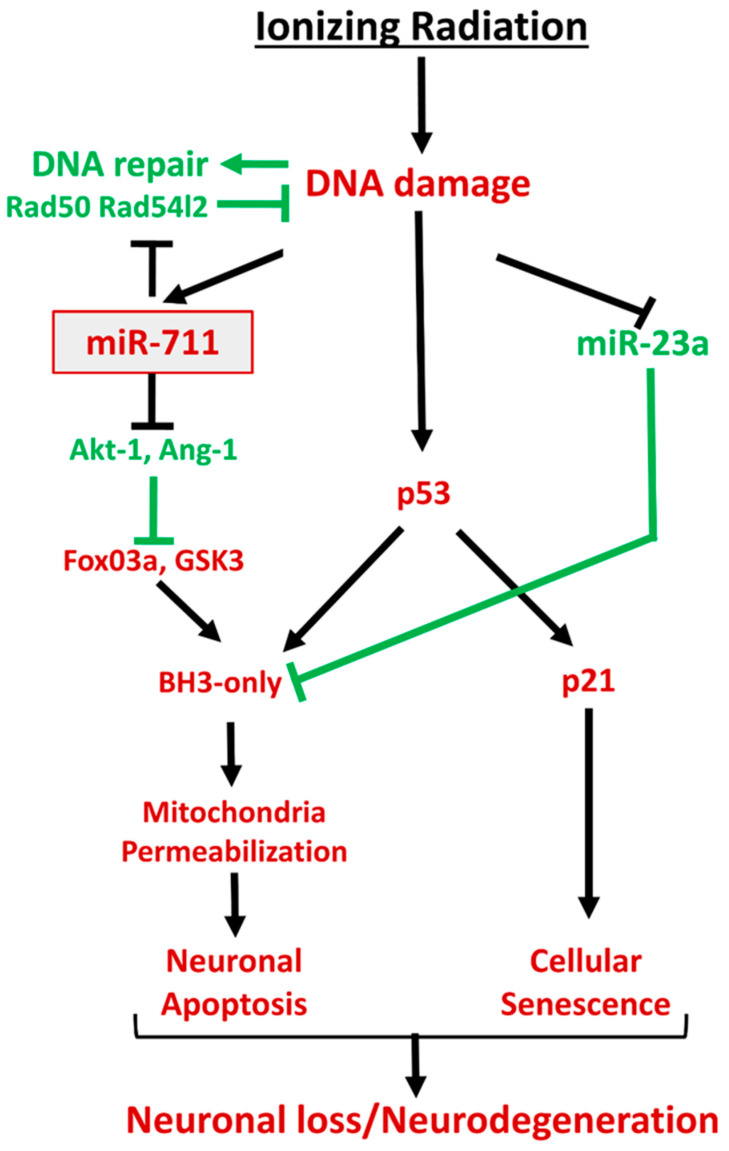
The schematic illustration of the role of miR-711 and miR-23a-3p on the IR-induced neuronal outcome. Pro-apoptotic events are shown in red, anti-apoptotic events are shown in green.

Our results show that irradiation induces regulated neuronal intrinsic apoptosis and senescence pathways and suggests that upregulation of miR-711 after irradiation contributes to neurodegeneration through multiple mechanisms: 1) inhibition of Akt-1/Ang-1 expression, leading to unhindered elevation of pro-apoptotic BH3-only molecules and 2) inhibition of Rad50 and Rad54l2, leading to impaired DNA-repair responses and increased ATM-dependent activation of p53-related pro-apoptotic as well as pro-senescence pathways. Furthermore, miR-711 inhibitors exert a concerted activation of DNA repair and survival mechanisms and inhibition of neuronal intrinsic apoptosis and senescence mechanisms and thus may be part of effective neuroprotective therapeutic interventions, which may stop the progression of neurodegeneration and promote neuronal survival. The scheme of investigated pathways is presented in Figure 10.

## 4. Methods

### 4.1. Animals and Radiation Delivery and Quality Control

Male C57BL/J6 mice (Jackson Labs, Bar Harbor, ME, USA) were irradiated as previously described [64]. Mice at approximately 10–12 weeks weighing ≥20 g were anesthetized by i.p. injection of 80–100 mg·kg^−1^ ketamine and 10–15 mg·kg^−1^ xylazine 15 min prior to radiation exposure to prevent them from moving out of the field. Mice were exposed to 10Gy of 320 kVp X-rays to the whole-brain (1.25 Gy·min−1, HVL = 1 mm Cu, Pantak 320 X-ray Irradiator, Precision X-ray Inc., North Branford, CT, USA). Control animals were anesthetized as described above to avoid anesthesia-related bias. A calibration quality check of the irradiator was performed each morning and again in between each radiation run. Port films (Gafchromic EBT2 dosimetry film, Ashland Inc., Covington, KY, USA) were acquired during each radiation run to verify that the brains of each mouse were properly positioned within the radiation field. All experiments were conducted in compliance with the Animal Use Protocol approved by the University of Maryland, School of Medicine, Office of Animal Welfare Assurance (OAWA), Institutional Animal Care and Use Committee (IACUC) protocol #0119015, approved 02/13/2019.

### 4.2. In Vitro Cell Culture

Pure neuronal cultures were used in this study to avoid interference from non-neuronal cells. Rat primary cortical neurons (RCNs) were derived from rat embryonic cortices as previously described [41]. Neurons were maintained in serum-free conditions using the B-27 Plus Supplement (Thermo Fisher Scientific, Waltham, MA, USA) according to the manufacturer’s protocol. RCNs were transfected with miR hairpin inhibitors using the Lipofectamine RNAiMAX Transfection Reagent (Invitrogen, Carlsbad, CA, USA) at 7 days in vitro according to the manufacturer’s protocol, with a few modifications. Briefly, half of the neuronal conditioned medium was removed from the culture and saved, RCNs were transfected, and the transfection medium was replaced with the saved half of the conditioned medium after 1 h. RCNs were transfected with miRIDIAN rat miR-711 hairpin inhibitor (miR-711 inhibitor) (IH-320669-01-0005), miRIDIAN microRNA hairpin inhibitor Negative Control (miR-ve inhibitor) (IN-001005-01-05), miRIDIAN rat miR-23a-3p mimic (C-320309-03-0005) and miRIDIAN microRNA Mimic Negative Control (CN-001000-01-05) (Dharmacon, Lafayette, CO, USA). The sequence of the miRIDIAN microRNA hairpin inhibitor Negative Control is based on *Caenorhabditis elegans* microRNAs and has minimal sequence identity in human, mouse and rat. Based on preliminary titration experiments, we chose a total final concentration of 50 nM for miR-inhibitors and -mimics. This concentration resulted in optimal transfection efficiency (~50%), was devoid of non-specific changes in non-targeted miRs and had no neurotoxic effects [27,41]. Moreover, the chosen concentration was associated with the highest neuroprotective effects (data not shown). For co-transfection with miR-23a-3p mimic and miR-711 inhibitor, the final concentration of each miR was 25 nM (50nM total). After culturing for seven days, in vitro RCNs were exposed to X-rays using a Pantak Seifert X-RAD X-Ray System (model number HS320, Precision X-Ray Inc) with energy settings 250 KeV and 13 mA. The 8Gy irradiation dose was chosen for treatment of RCN as the lowest dose which causes significant neuronal cell death.

In some experiments, downstream signaling pathways were targeted using the following inhibitors: Akt inhibitor (1L6-hydroxymethyl-chiro-inositol-2-(R)-2-O-methyl-3-O-octadecyl-sn-glycerocarbonate; Calbiochem, Billerica, MA, USA), which selectively inhibits Akt (PKB; IC50 of 5.0 μM) and moderately inhibits PI3-K activity (IC50 = 83.0 μM) and Wortmannin (Cell Signaling, Technology Inc., Danvers, MA, USA), which inhibits PI3 kinase. To confirm the neuroprotective effect of Ang-1 and Akt in IR-induced neuronal cell death, RCNs were treated with a Carrier Free Recombinant Human Angiopoietin-1 Protein (Ang-1) (R&D Systems Inc.cat# 923-AN-025/CF, Minneapolis, MN, USA) that shares 97% amino acid sequence identity with mouse and rat Angiopoietin-1 to final concentrations 50, 100, 200 and 400 ng/mL alone or with 200 ng/mL of recombinant Ang-1 and 6.25uM Akt inhibitor or 25 nM of Wortmannin.

### 4.3. Immunohistochemistry

RCNs were fixed and co-stained as described previously [65]. For immunocytochemistry, we transfected primary cortical neurons with miR-711 hairpin inhibitor or negative control (non-targeting) hairpin inhibitor 1 h before IR (8Gy) on DIV 7 in 24-well plates with coverslips. After 30 min, 6 h or 24 h, RCNs on coverslips were fixed for 10 min in 4% paraformaldehyde/PBS and then co-stained with a 1:200 dilution of Cell Signaling’s γ-H2A.X (CST #9718) antibody and a 1:400 dilution of Millipore’s Milli-Mark™ Pan Neuronal Marker (data not shown) in 10% goat serum (Gemini Bio-Products, West Sacramento, CA, USA) overnight at 4 °C. Wells were incubated the next day with goat-derived secondary antibody (Life Technologies, Fisher Scientific, Hampton, NH, USA), followed by 4’,6-diamidino-2-phenylindole (DAPI, Sigma-Aldrich, St. Louis, MO, USA) (0.5 µg/mL in saline). Imaging was performed using an Orca^®^-Flash4.0LT Digital CMOS camera( Hmamamatsu, Hamamatsu, Japan) mounted on a Nikon Eclipse Ni-E microscope with a Nikon Plan Apo 60X/1.40 OIL WD objective( Nikon, Tokyo, Japan). Exposure times and laser power settings were optimized to maximize signal intensity in controls without oversaturating signal in higher-intensity samples and maintained constant for all images with the same staining. The analysis was done using Nikon’s NIS-Elements software (version 5.11.01) and the “General Analysis” tool. For each treatment, between four and seven separate/non-overlapping fields were selected and a series of 2048 × 2044 pixel images was acquired at a resolution of 16 bits with a z-distance of 0.3 µm. Each set of images for a given field was then used to generate a single image via maximum intensity projection. These maximum intensity projections were analyzed with settings as follows:

For γ-H2A.X-stained samples: DAPI was used to identify nuclei by Rolling Ball Correction (radius 8.02 µm) → Local Contrast (Size 35, Power 95%) → Threshold → Smooth (2×), Fill holes, Separate (2×) → Object Area filtering (5–120 µm^2^), Morpho Separate Objects (2×). Foci were then identified via γ-H2A.X staining by Rolling Ball Correction (radius 1.52 µm) → Bright Spot Detection (Typical Diameter = 0.399 µm, Contrast = 713.6).

For 53BP1-stained samples: DAPI was used to identify nuclei as described above. Foci were then identified via 53BP1 staining by Rolling Ball Correction (radius 1.95 µm) → Bright Spot Detection (Typical Diameter = 0.399 µm, Contrast = 346.7) → Remove Dark Objects.

Analysis was performed on all nuclei containing foci thus identified, and the number of foci of γ-H2A.X /53BP1 within each nucleus was quantified and plotted for all fields together as an unbinned cumulative frequency distribution [65].

### 4.4. RCNs Irradiation

After culturing for seven days, in vitro RCNs were exposed to 150 kV X-rays (HVL ~ 1.5 mm Cu, 13 × 13 cm2, 29.2 cm source-to object distance, 2.3 Gy/min) using a Seifert X-ray system (model number HS320). The irradiator is calibrated following the American Association of Physicists in Medicine (AAPM) Task Group (TG) [66] protocol for 40–300 kVp X-ray source calibration, using a national Institute of Standards and Technology (NIST)-traceable PTW TN30013 Farmer-type ionization chamber and PTW T10010 Unidose electrometer (PTW, Freiburg, Germany). The 8Gy irradiation dose was chosen as the lowest dose, which causes significant neuronal cell death.

### 4.5. Cell Death Assays

As TUNEL staining is not a reliable method to examine apoptotic DNA fragmentation after IR since IR induces the DNA double-strand breaks that TUNEL staining assays [67], LDH release assays were used to examine neuronal cell death. Cell death was measured using the CytoTox 96^®^ Non-Radioactive Cytotoxicity Assay, as previously described [41] or LDH-Glo^TM^ Cytotoxicity Assay (J2380 Promega, Madison, WI, USA) with some modifications: by combining 10 µL of media from a 96-well plate well with 10 µL of Detection Enzyme and Reductase Substrate, premixed just before assay in the proportion recommended in the protocol and then diluted 1:10 in LDH Storage Buffer (also prepared as recommended in the protocol). To induced maximum LDH, release 10 µL of 9% (*v*/*v*) Triton^®^ X-100(Sigma Aldrich) was added to the wells to permeabilize all cells (100% cell death). Luminescence was measured after 1 h incubation in the dark in a BioTek Synergy HT Plate Reader using Gen5™ software(BioTek, Vinuski, VT, USA). Each treatment/time point reflects six replicates for all assays performed in RCNs cultured in 96-well plates.

### 4.6. RNA-Interacting Protein Immunoprecipitation (RIP) Using AGO2-Specific Antibodies

miRNA–mRNA pairs were purified as previously described [23]. One strand of the mature miR binds to Argonaute (Ago) proteins to form the RNA-induced silencing complex (RISC), and the miR acts as a template for recognition and cleavage of complementary mRNA. miRNA–mRNA target pairs can be purified by immunoprecipitation of the RISC components to confirm mRNA targets. Ago2 immunoprecipitation was performed as previously described [23] with a few modifications. Briefly, RCNs were suspended in 500 µL of lysis buffer: 150 mM KCl, 25 mM Tris-HCl (pH 7.4), 5 mM EDTA, 0.5% IGEPAL CA-630, 5 mM DTT, RNase Inhibitor (Thermo Fisher Scientific, Waltham, MA, USA N8080119) to a final concentration of 10 U/mL and protease inhibitor and phosphatase inhibitor (2, 3) cocktails (Sigma-Aldrich, St. Louis, MO, USA) at 4 °C for 20 min., and the cell lysates were separated by centrifugation at 12,000 × g for 20 min at 4 °C. A pre-clearing step was added to reduce non-specific binding. Ten microliters of Rabbit (DA1E) mAb IgG XP^®^ Isotype Control (# 3900 Cell Signaling) was added to lysates, and the mixtures were rotated for 1 h at 4 °C. Fifty microliters of protein A/G UltraLink Resin (Thermo Scientific, Waltham, MA, USA) was then added to lysates, and the mixture was rotated for 30 min at 4 °C. After incubation, beads were removed by centrifugation at 1000 × g at 4 °C for 5 min. The supernatant was used for immunoprecipitation. One part of the lysate from each sample was used for RNA isolation and qPCR analysis for levels of GAPDH in the Inputs. A volume of 50 µL of protein A/G UltraLink Resin (Thermo Scientific, Waltham, MA) and 20 µL of Argonaute 2 (Ago2) antibody (Cell Signaling) was added to 400 µL of cell lysate (in a final 1 mL mixture filled with lysis buffer), and the mixture was rotated for 4 h at 4 °C. The beads were washed three times with 1 mL lysis buffer to remove non-specific binding. RNAs bound on the beads were extracted via the miRNeasy Kit (Qiagen). miR and gene expression was analyzed by qPCR as described below. The levels of mRNA and miR-711 were normalized to GAPDH and U6 snRNA (001973 Applied Biosystems, Foster City, CA, USA) levels in Inputs, respectively. We controlled the variations in the Inputs by normalizing levels of mRNAs to GAPDH and miR-711 to U6 snRNA. We confirmed the specificity of Ago2/RISC IPs by using normal rabbit IgG (#3900 Cell Signaling) for negative control IPs. qPCR analysis confirmed that the levels of target mRNAs and miR-711 in negative control IPs were more than 17 times lower than in IPs with control (non-irradiated) samples.

### 4.7. RNA Isolation

Total RNA was isolated using the Direct-zol RNA Kits (Zyme Research, Irvine, CA, USA). During the process of isolation, samples were treated with RNase-free DNase (Qiagen, Hilden, Germany) to digest DNA contamination of the samples according to the manufacturer’s protocol.

### 4.8. qPCR

Verso cDNA Kit (Thermo Scientific, Waltham, MA, USA) was used to synthesize cDNA from purified total RNA as described previously [23]. Water was used instead of RNA in the negative control no-template reaction. Water was used instead of enzyme in the negative control no-enzyme reactions. Quantitative real-time PCR was performed by using cDNA TaqMan Universal Master Mix II (Applied Biosystems, Foster City, CA, USA). TaqMan Gene Expression assays for the following genes were used for rat GAPDH (Rn01775763_g1, Mm99999915_g1), p21 (Rn01427989_s1), PUMA (Rn00597992_m1), Noxa (Rn01494552_m1), Bim (Rn00674175_m1), p53 (Rn00755717_m1), Akt1 (Rn00583646_m1), Ang-1 (Rn01504818_m1), Rad50 (Rn00573802_m1), Rad54l2 (Rn01446690_m1), Mecp2 (Rn01529606_g1), Hdac1(Rn01519308_g1), Hdac4 (Rn01427040_m1), Hdac2 (Rn01193634_g1), U6 snRNA (001973); for mouse mmu-miR-711 (001646), Akt1 (Mm01331626_m1), Ang-1 (Mm00456503_m1), p21 (Mm04205640_g1), synapsin I (Syn1) (Mm00449772_m1), Bim (Mm00437796_m1), glial fibrillary acidic protein (GFAP) (Mm01253033_m1) and integrin alpha M (Itgam, CD11b) (Applied Biosystems, Foster City, CA, USA). Reactions were amplified and quantified using a Quant Studio 5 system and the corresponding software (Applied Biosystems, Foster City, CA, USA). Reactions were performed in duplicates. Water was used instead of cDNA in the negative no-template control reactions. No-template and no-enzyme negative controls from the reverse transcription step were used to eliminate false-positive results. Gene expression was normalized to GAPDH, and the relative quantity of mRNAs was calculated based on the comparative Ct method [68].

### 4.9. miR Reverse Transcription and qPCR

Quantitative real-time PCR was used to measure the expression of mature miR-711. A unit of 10 ng of total RNA was reverse-transcribed using TaqMan miRNA Reverse Transcription Kit (Applied Biosystems, Foster City, CA, USA) with miRNA-specific primers. Reverse transcription reaction products (1.5 µL) were used for qPCR as described above. TaqMan Gene Expression assays for the following miRs were used: rno-miR-711 (241136 mat), pri-mir-711 (Rn04229705_pri), and U6 snRNA (001973) (Applied Biosystems, Foster City, CA, USA). miRs levels were normalized to U6 snRNA (001973, specific for both rat and mouse).

### 4.10. Isolation of Neurons from the Mouse Brain and qPCR

Part of the cortex and the hippocampus were separately dissected from the brain for total RNA isolation. The parietal regions of the left and right cortices as well as the entire left and right hippocampi were used for RNA isolation. The remaining cerebral hemispheres were used for neuronal isolation by MACS separation technology (Miltenyi Biotec, Bergisch Gladbach, Germany). Briefly, brain tissues were rapidly microdissected, and a single-cell suspension was prepared using enzymatic digestion (Adult Brain Dissociation Kit; Miltenyi Biotec) in combination with a gentle MACS™ Octo Dissociator. The cells were incubated with Non-Neuronal Cells Biotin-Antibody Cocktail (Miltenyi Biotec) and loaded onto LS columns (Miltenyi Biotec) placed in the magnetic field of a MACS separator. The negative fraction (flow through–neurons) was collected, and the column was washed three times with D-PBS/BSA buffer (Miltenyi Biotech). Non-neuronal positive cells were eluted by removing the magnetic field. The purity of neuronal cell isolation was confirmed by qPCR with pooled neuronal and non-neuronal samples from each group being probed with primers specific to the astrocyte marker—GFAP—and the microglia marker—CD11b. The average levels of GFAP and CD11b mRNAs were approximately 25 times and 100 times lower, respectively, in isolated neuronal cells compared to non-neuronal cells from the same isolation (data not shown).

Total RNA was isolated using the Direct-zol RNA Microprep kit (Zymo Research) according to the manufacturer’s protocol. Two hundred nanograms of total RNA was used for cDNA syntheses as described above. Gene-specific pre-amplification was used to enhance the amount of input material for qPCR. An aliquot of each cDNA sample equivalent to 20 ng RNA was used for pre-amplification with TaqMan Universal Master Mix II (Applied Biosystems, Foster City, CA, USA). The total volume of pre-amplification was 20 μL for each sample. The reaction contained 5 μL of the master mix, 2 μL of cDNA, 5 μL of pooled TaqMan Gene Expression assays and 8 μL of water. The following temperature protocol was used: 50 °C for 2 min, 95 °C for 10 min, followed by 10 cycles, respectively, at 95 °C for 15 s and 60 °C for 1 min. Water was used instead of cDNA in the negative control reaction. The pre-amplified cDNA was diluted 10 times. Two microliters of diluted pre-amplified cDNA was used for each qPCR reactions. qPCR was performed as described above. 

### 4.11. ChIP Assay

ChIP assays were performed as described previously [65] by EpiQuik™ Chromatin Immunoprecipitation (IP) Kit (Epigentek, Farmingdale, NY, USA) according to the manufacturer’s instructions. Briefly, 5 × 10^6^ RCNs were crosslinked using 10 mL of phosphate-buffered saline (PBS) containing 1% formaldehyde (final concentration) and used for ChIP. Chromatin was sheared to fragments ranging from 200 to 600 bp by a Bioruptor sonication device (Diagenode, Denville, NJ, USA). Immunoprecipitation was performed for 90 min with 2 µg of p53 antibodies (D2H9O, Cell Signaling Technology). Normal rabbit IgG (#3900 Cell Signaling Technologies) and anti-RNA polymerase II antibody (Epigentek, Farmingdale, NY, USA) were used as negative and positive IP controls. There was 5.2 times less PCR product for the negative control IP and 6.5 more PCR product for the positive control IP compared to PCR product for the average IP sample. p53 occupancy of the Noxa promoter was analyzed by qPCR (described above). The predicted p53-binding site in the promoter region of the rat Noxa gene is located at 62 914 518 nt on chromosome 18 (NC_005117.4). The sequence of the p53 binding site is: 5′-CGGCTTGCCCCGGCAAGTTG-3′ (62,174,513-62,174,533 nt on Rat chromosome 18). For qPCR, we designed the following qPCR assay: forward primer: 5′- CTTCCCTCCCACCTTCGTTT-3′; reverse primer: 5′- GCCGGCTCTCGGGTTTTAT-3′; probe /56-FAM/AGCTTTACT/ZEN/TCTCTTCGCTCCCGC/3IABkFQ/; oligonucleotides were synthesized by Integrated DNA Technologies, Inc. (Coralville, IA, USA) qPCR reactions were performed in duplicate in a total volume of 20 μl. The final concentration of primers was 750 nM, and 250 nM for the probe. The PCR profile consisted of one cycle at 50 °C for 2 min and 95 °C for 10 min, followed by 40 cycles at 95 °C for 15 s, 55 °C for 30 s and 60 °C for 1 min. The efficiency of the reaction was determined by qPCR of serially diluted samples. The efficiency of reaction for this assay was 77.1%. The level of amplification in the IP samples was normalized to Input samples and the relative quantity of p53 occupancy was calculated by using the following equation: Fold difference = ((E target)^ΔCt target)/((E normalizer)^ΔCt normalizer); E = efficiency of qPCR. This equation was derived from the comparative Ct method [69] by Applied Biosystems (Foster City, CA, USA).

### 4.12. Cell Lysates Preparation and Western Blot

Whole-cell extracts and Western blot were prepared/performed as previously described [70]. Chemiluminescence was captured on a ChemiDoc Touch Imaging System (Bio-Rad, Hercules, CA, USA), and protein bands were quantified by densitometric analysis using Image Lab Imaging Software (Bio-Rad, Hercules, CA, USA). The data presented reflect the intensity of the target protein band relative to the control and were normalized based on the intensity of the endogenous control (β-actin unless otherwise stated) for each sample (expressed in arbitrary units).

### 4.13. Antibodies

The following antibodies were used in this study: Histone H2A.X (ab11175; Abcam); AIF (sc-13116), cytochrome c (sc-13560; Santa Cruz Biotechnology, Dallas, TX, USA); ((Ph-H2A.X(Ser139)-γ-H2A.X) (#9718), phosphorylated ataxia telangiectasia mutated kinase ((Ph-ATM (Ser1981)-Ph-ATM), (Phospho-ATR(Ser428)-Ph-ATR) (#2853), Cleaved Caspase-3 (#9661), PARP (#9542), (phospho-p53(Ser15)-Ph-p53) (#9284), PUMA (#14570), p53 (#2524), post-synaptic density protein 95 (PSD95) (#3450) (Cell Signaling); GAPDH (ADI-CSA-335) and α-fodrin (BML-FG6090; Enzo Life Sciences, Inc., Farmingdale, NY, USA); β-actin (A1978; Sigma, St. Louis, MO); phospho-ATM (05–740 Millipore, Burlington, MA, USA); p21 (556430 BD Biosciences, San Jose, CA, USA). We observed two bands in immunoblots with antibodies against GAPDH. The size of the top band was ~37kDa, which matches the size of GAPDH according to the supplier. Cytochrome c levels were normalized to the top ~37kDa band of GAPDH. The double bands of GAPDH in the cytosolic fraction have been previously reported [13,38]. We observed three bands in immunoblots with antibodies against phospho-ATR (Ser428) for all samples and conditions. The size of the middle band was ~300 kDa. According to the supplier, the 300kDa band is specific for phospho-ATR(Ser428), and this band was used for our analyses.

### 4.14. Subcellular Fractionation

Subcellular fractionation was performed as described previously [23]. RCNs were harvested and washed in ice-cold phosphate-buffered saline. The cell suspension was centrifuged at 500 × g for 15 min at 4 °C. The cell pellet was resuspended for 10 min on ice in the digitonin lysis buffer (20 mM HEPES, pH 7.4, 80 mM KCl, 1 mM EDTA, 1 mM EGTA, 1 mM DTT, 250 mM sucrose, 200 μg/mL digitonin and protease inhibitor and phosphatase inhibitor (2, 3) cocktails (Sigma-Aldrich, St. Louis, MO). Cells were passaged 20 times through a 22G needle. The lysate was centrifuged at 1000 × g for 5 min at 4 °C to pellet the nuclei. The supernatant was transferred to a new tube and centrifuged again at 12,000 × g for 10 min at 4 °C to precipitate the mitochondria. The resulting supernatant, representing the cytosolic fraction, was recovered. Nuclear and mitochondrial lysates were prepared in RIPA buffer (Teknova) with Protease Inhibitor Cocktail (Sigma-Aldrich, St. Louis, MO). All steps were performed on ice. β-actin is a cytoskeleton protein, and that is why it was used to normalize Western blot data from total lysates. GAPDH is a cytoplasmic marker and was therefore used to normalize Western blot data from cytoplasmic fractions.

### 4.15. Statistical Analysis and General Methods

Samples for all analyses were randomized by giving them numbers without a description of the treatment. All in vitro experiments were repeated at least three times with similar results. All data passed the normality test (alpha = 0.05). All statistical analyses were performed using Graphpad Prism 7. One-way ANOVAs with Tukey post hoc tests were used to analyze Western blot, qPCR, ChIP, LDH assays, except for cases when only two groups were compared. In this case, we used a one-tailed t-test. Immunocytochemistry data were analyzed using the Kruskal–Wallis test followed by Dunn’s post-hoc analysis. In all graphs, data represent the mean ± SEM. 

### 4.16. Experimental Setup

#### 4.16.1. Experiment 1

We examined the activation of apoptosis and senescence pathways by qPCR not only in the cortex and hippocampus but also in purified neurons isolated from cortical and sub-cortical regions of adult mouse brains after 10Gy whole-brain irradiation. We also analyzed IR-induced changes in miR-711 levels as well as alterations in the expression of miR-711 targets. *n* = 6 for each group for tissues, *n* = 5 for each group for isolated neurons.

#### 4.16.2. Experiment 2

Using Western blot, we measured the levels of Ph-ATM(Ser1981) and γ-H2A.X to investigate whether X-ray irradiation induced the activation of DNA damage responses and the levels of total p53, Ph-p53(Ser15) and p21 to evaluate the activation of p53 pathways at 30 min and 24 h after 2, 8 and 32 Gy irradiation, compared to non-irradiated controls. Levels of phosphorylated proteins were normalized both to β-actin and to the parent protein. qPCR was used to analyze expression of p21 at the same time points after IR. The experiment was repeated 3 times, *n* = 3/group.

#### 4.16.3. Experiment 3

In neuronal apoptosis, activated p53 upregulates the expression of pro-apoptotic members of the Bcl-2 family. To examine the effect of IR-induced p53 activation on Noxa expression, we used ChIP with p53 antibodies, followed by qPCR. Neurons were collected 6 h after 8Gy irradiation to examine p53 occupancy on a Noxa promoter region. *n* = 3/group, with 2 technical replicates. In order to evaluate expression of the Bcl-2 family members, neurons were collected 30 min, 6 h and 24 h after 2, 8 and 32Gy irradiation, and total RNA was used for qPCR analysis. We also examined the expression of pro-survival Bcl-2 family members such as B-cell lymphoma-extra-large (Bcl-XL) and B-cell lymphoma 2 (Bcl-2). *n* = 5 for all groups, with 2 technical replicates per sample.

#### 4.16.4. Experiment 4

To investigate the effect of IR on neuronal cell death, we treated neurons with 2, 8 and 32Gy. Twenty-four hours later, LDH release was measured. The experiment was repeated 2 times with 6 biological replicates per group. Next, we checked for markers of neuronal apoptosis and neurodegeneration 30 min and 24 h after 2, 8 and 32Gy irradiation by Western blot. The experiment was repeated 3 times, *n* = 3/group.

#### 4.16.5. Experiment 5

Previously, we demonstrated that neuronal-injury-induced DNA damage causes upregulation of miR-711 and downregulation of its validated anti-apoptotic targets Akt and Ang-1. We examined the effect of IR on the expression of miR-711, Akt and Ang-1 mRNA. The experiment was repeated 3 times. *n* = 3 for all groups, with 2 technical replicates per sample. Next, we used qPCR after RNA-interacting protein immunoprecipitation (RIP) with Ago2 antibodies to confirm the role of miR-711 in silencing Akt and Ang-1 through the RISC (RNA-induced silencing complex). The experiment was repeated 2 times. *n* = 3/group, with 2 technical replicates.

#### 4.16.6. Experiment 6

To test the importance of miR-711 in causing radiation-induced downregulation of Akt and Ang-1, RCNs were transfected with miR-711 hairpin inhibitor or miR-ve inhibitor 1 h before exposure to 8Gy IR. Levels of miR-711, Akt and Ang-1 mRNAs were measured at different time points by qPCR. *n* = 4 for the control group; *n* = 5 for all other groups. The experiment was repeated 3 times, with 2 technical replicates. To confirm the neuroprotective effect of Ang-1 and Akt in IR-induced neuronal cell death, RCNs were treated with recombinant Ang-1 or with recombinant Ang-1 and Akt inhibitor or Wortmannin. The experiment was done once with 6 biological replicates. Next, we used qPCR after RIP with Ago2 antibodies to confirm the role of miR-711 in silencing Akt and Ang-1 through the RISC. The experiment was repeated two times. *n* = 3/group, with 2 technical replicates.

#### 4.16.7. Experiment 7

We examined the ability of miR-711 inhibitor to attenuate neuronal apoptosis by evaluating apoptotic markers at various time points after irradiation (+/− inhibitor) via Western blot. The experiment was repeated three times, *n* = 3 per group. We also used qPCR to analyze the expression of Bcl-2 family members. The experiment was repeated three times, *n* = 3/group, with 2 technical replicates. Next, we examined the effect of miR-711 inhibition on pro-apoptotic Bcl2 member-induced MOMP and cytosolic release of AIF-1 and cytochrome c by Western blotting after subcellular fractionation. The experiment was repeated two times, *n* = 3 per group. We examined the prolonged effect of IR and miR-711 inhibition on neuronal cell death by measuring LDH release 24 h and 48 h after irradiation, *n* = 6 for each group.

#### 4.16.8. Experiment 8

Next, we tested the effect of miR-711 inhibition on IR-induced DNA damage and p53 activation at various time points by Western blot. The experiment was repeated 3 times, *n* = 3 per group. qPCR was used to analyze expression of p53 and p21 at the same time points after IR. The experiment was repeated three times, *n* = 5 for control, *n* = 3 for all other groups, with 2 technical replicates.

#### 4.16.9. Experiment 9

Since miR-711 inhibitor attenuates IR-induced increase of DNA damage markers, we used Gene Ontology (GO) enrichment analysis [71], which revealed that some of the TargetScan-predicted [72] miR-711 targets may be involved in DNA damage response/repair and modification including Methyl-CpG-binding protein 2 (Mecp2), ERCC excision repair2 (Ercc2), histone decetylases (HDACs) 1, 2, 4, Rad50 and Rad54l2. To test the suggestion that these genes may be targeted by IR-induced miR-711, we transfected RCNs with miR-711 inhibitors or miR-ve inhibitors before exposure to 8Gy and analyzed expression of these genes by qPCR. Our results led us to further investigate the role of miR-711 in the regulation of Rad50 and Rad54l2 expression. After RIP using Ago2 antibodies, levels of miR-711, Rad50 and Rad54l2 mRNAs in the RISC were measured by qPCR. The experiment was repeated two times, *n* = 3/group, with 2 technical replicates. Next, we examined the effect of miR-711 inhibition on levels of miR-711, Rad50 and Rad54l2 in the RISC via the same technique, *n* = 3 for each group, with 2 technical replicates. 

We confirmed the effect of miR-711 inhibition on DNA damage repair mechanisms by quantifying γH2A.X and 53BP1 foci formation and progression after 8Gy irradiation. This experiment was repeated twice; we quantified between four and seven separate/non-overlapping fields per treatment with an average of 202 cells per field. 

#### 4.16.10. Experiment 10

We have recently reported that IR downregulates the pro-survival miR-23a-3p [35]. We examined the effect of miR-711 inhibition on the level of neuronal miR-23a-3p after IR exposure to investigate interactions between miR-23a-3p and miR-711 neuroprotective mechanisms. We also examined the synergistic effects of miR-711 inhibitor and miR-23a-3p mimic after IR exposure.

## Supplementary Materials

Supplementary materials can be found at https://www.mdpi.com/1422-0067/21/15/5239/s1. Figure S1. qPCR quantification of MECP2, Ercc2, HDAC1, HDAC2 and HDAC4 (E). Figure S2. miR-711 inhibition did not alter the IR-induced down-regulation of miR-23a-3p.
