# Supplementary material for: Irradiation-Induced Upregulation of miR-711 Inhibits DNA Repair and Promotes Neurodegeneration Pathways
Source: Int J Mol Sci. 2020 Jul 23;21(15):5239. doi: 10.3390/ijms21155239 (PMC7432239; doi:10.3390/ijms21155239)
Supplement: Supplementary file 1 [file ijms-21-05239-s001.pdf]

International Journal of
Molecular Sciences

Supplementary Figures

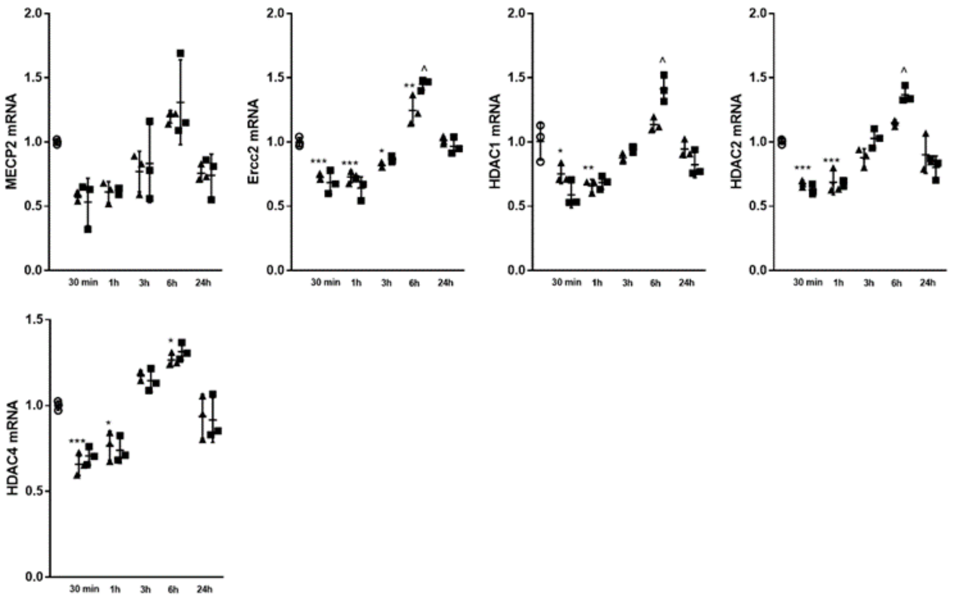

Figure S1. qPCR quantification of MECP2, Ercc2, HDAC1, HDAC2 and HDAC4 (E). Mecp2 mRNA[F(10,22) = 7.046, no significant changes], Ercc2 mRNA [F(10,22) = 50.29, $p = 0.0006$ at 30m, $p = 0.0008$ at 1h, $p = 0.0406$ at 3h, $p = 0.0024$ at 6h after IR with miR-ve inhibitor compared to control; for miR-711 inhibitor compared to miR-ve inhibitor, $p = 0.0167$ at 6h], HDAC 1 mRNA[F(10,22) = 25.88, $p = 0.0298$ at 30m, $p = 0.0012$ at 1h after IR with miR-ve inhibitor compared to control; for miR-711 inhibitor compared to miR-ve inhibitor, $p = 0.0130$ at 6h], HDAC2 mRNA[F(10,22) = 30.71, $p = 0.0006$ at 30m, $p = 0.0009$ at 1h after IR with miR-ve inhibitor compared to control; for miR-711 inhibitor compared to miR-ve inhibitor, $p = 0.0352$ at 6h], HDAC 4 mRNA[F(10,22) = 28.71, $p = 0.0006$ at 30m, $p = 0.0309$ at 1h, $p = 0.0101$ at 6h after IR with miR-ve inhibitor compared to control]. N = 3/group for all groups, *$p < 0.05$, **$p < 0.01$, ***$p < 0.001$ vs. control; ^$p < 0.05$, vs. corresponding 8Gy + miR-ve inhibitor group.

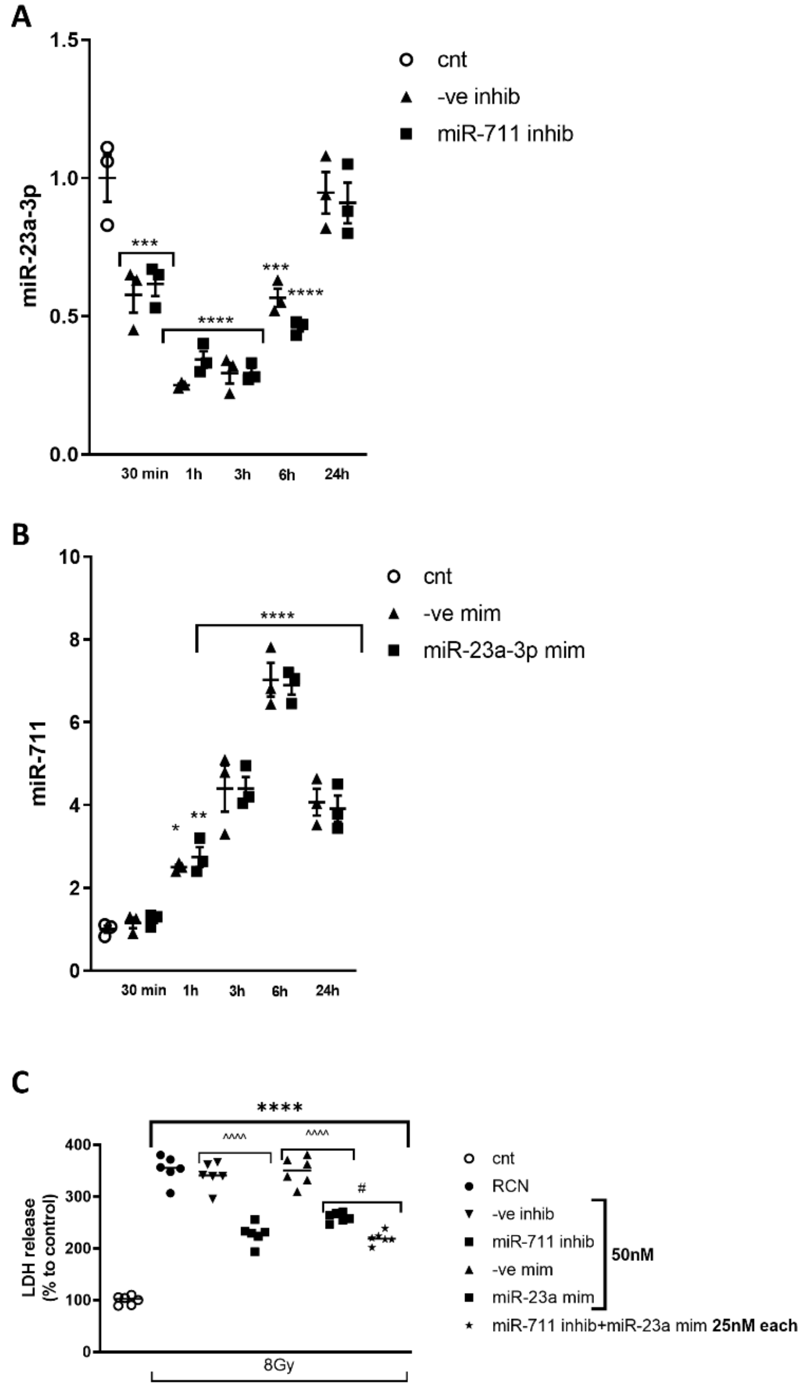

Figure S2. miR-711 inhibition did not alter the IR-induced down-regulation of miR-23a-3p. (**A**) RCNs were transfected with miR-711 inhibitor and miR-ve inhibitor 1h before exposure to 8Gy. qPCR quantification of miR-23a-3p [$F(10,22) = 29.56$, $p < 0.0003$ at 30min after IR for miR–ve inhibitor, $p < 0.001$ for miR-711 inhibitor; $p < 0.0001$ for miR–ve inhibitor and miR-711 inhibitor at 1, 3 and 6h after IR for both compared to non-irradiated control. (**B**) RCNs were transfected with miR-23a-3p mimic and miR-ve mimic 1h before exposure to 8Gy. qPCR quantification of miR-23a-3p [$F(10,22) = 54.32$, $p < 0.0379$ at 1h after IR for miR–ve mimic, $p < 0.0099$ for miR-23a-3p mimic; $p < 0.0001$ for miR–ve inhibitor and miR-711 inhibitor at 3, 6 and 24h after IR for both compared to non-irradiated control. N = 3/group for all groups, *$p < 0.05$, **$p < 0.01$, ***$p < 0.001$, ****$p < 0.0001$ *vs*. control. (**C**) LDH was measured 24h after irradiation [$F(6,35) = 132.4$, $p < 0.0001$ at 24h after IR compared to non-irradiated

control; $p < 0.0001$ for miR-711 inhibitor compared to miR-ve inhibitor, $p < 0.0001$ for miR-23a-3p mimic compared to miR-ve mimic, inhibitor, $p < 0.0169$ for miR-23a-3p mimic (25nM) + miR-711 inhibitor (25nM) compared to miR-23a-3p mimic (50nM). N = 6/group, ****$p < 0.0001$vs. control, ^^^^$p < 0.0001$ vs. miR-ve mimic and inhibitor, #$p < 0.05$ vs miR-23a-3p mimic.

© 2020 by the authors. Submitted for possible open access publication under the terms and conditions of the Creative Commons Attribution (CC BY) license (http://creativecommons.org/licenses/by/4.0/).
